# Direct effect of 2‐palmitoyl glycerol on promotion of gamma aminobutyric acid synthesis in normal human fetal‐derived astrocytes

**DOI:** 10.1002/2211-5463.13649

**Published:** 2023-05-24

**Authors:** Misato Tsuboi, Yoshitaka Nakamura, Hiroshi Sakuma

**Affiliations:** ^1^ Food Microbiology and Function Research Laboratories, R&D Division Meiji Co., Ltd. Tokyo Japan; ^2^ Department of Pediatric Neurology Niigata University Graduate School of Medical and Dental Sciences Japan; ^3^ Department of Brain and Neuroscience Tokyo Metropolitan Institute of Medical Science Japan

**Keywords:** 1,3‐dioleoyl‐2‐palmitoylglycerol, astrocytes, brain development, breast milk, gamma aminobutyric acid metabolism

## Abstract

Breast milk contains constituents, such as 1,3‐dioleoyl‐2‐palmitoylglycerol (OPO), that are beneficial for infants. Herein, we hypothesized that 2‐palmitoyl glycerol (2‐PG), a derivative of OPO, is advantageous to infants' development. Gamma aminobutyric acid (GABA) is a major neurotransmitter involved in neural development. Although GABA is generally known to be produced in neurons, astrocytes can also produce it in immature brains. In this study, we used expression analysis techniques to show that 2‐PG upregulates the mRNA and protein expression of glutamate decarboxylases (GAD1 and GAD2) in normal human fetal‐derived astrocytes. Our data suggest that 2‐PG promotes GABA synthesis in astrocytes, which may contribute to brain development because GABA is involved in neural development in the developing brain. This may help to elucidate the mechanism by which breast milk affects infant brain development.

## Graphical abstract

2‐palmitoyl glycerol (2‐PG) significantly upregulated the mRNA of GABA‐related genes including glutamate transporter (blue arrow) and protein expression of glutamate decarboxylases (red arrow) in normal human fetal‐derived astrocytes. Our data suggest that 2‐PG promotes GABA synthesis in astrocytes, which may contribute to brain development because GABA is involved in neural development in the developing brain.

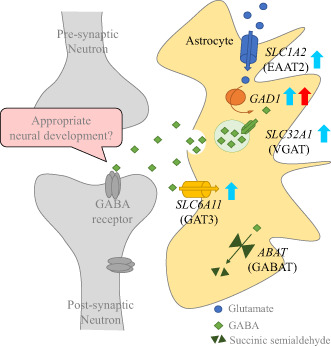

**Abbreviations**
2‐AG: 2‐arachidonoylglycerol
2‐PG: 2‐palmitoyl glycerol
3‐PG: 3‐palmitoyl glycerol
ABAT: 4‐aminobutyrate aminotransferase
ACS: acyl‐coA synthase
ADCY8: adenylate cyclase 8
ADRBK1: adrenergic, beta, receptor kinase 1
ADRBK2: beta‐adrenergic receptor kinase 2
ANOVA: analysis of variance
BGT1: Na(+)/Cl(−) betaine/GABA transporter
BSA: bovine serum albumin
CACNA: calcium voltage‐gated channel subunit alpha
CD36: cluster of differentiation 36
DMEM: Dulbecco's Modified Eagle's Medium
DNA: deoxyribonucleic acid
EAAT: excitatory amino acid transporter
ELISA: Enzyme‐Linked Immuno Sorbent Assay
FABPs: fatty acid‐binding proteins
FAT: fatty acid translocase
FATPs: fatty acid transportation proteins
FFA: free fatty acid
GABA: gamma aminobutyric acid
GABARB: gamma aminobutyric acid receptor subunit beta
GABRA: gamma aminobutyric acid receptor subunit alpha
GAD: glutamate decarboxylases
GADPH: glyceraldehyde‐3‐phosphate dehydrogenase
GAT: GABA transporter
GCOS: gene chip operating system
GNG10: G protein subunit gamma 10
GNGT1: G protein subunit gamma transducin 1
GRIA: glutamate ionotropic receptor AMPA
GRIN2A: glutamate ionotropic receptor NMDA type subunit 2A
GRIN3B: glutamate receptor ionotropic, NMDA 3B
GRK3: G protein‐coupled receptor kinase 3
GRM: glutamate metabotropic receptor
GRM1: glutamate metabotropic receptor 1
HAP1: Huntingtin‐associated protein 1
HOMER3: Homer scaffold protein 3
HRP: horseradish peroxidase
ITPR3: inositol 1,4,5‐trisphosphate receptor, type 3
KEGG: Kyoto Encyclopedia of Genes and Genomes
Mfsd2a: major facilitator superfamily domain‐containing protein
NHA: normal human fetal‐derived astrocytes
OPO: 1,3‐dioleoyl‐2‐palmitoylglycerol
PA: palmitic acid
PBS: phosphate‐buffered saline
PBS‐T: phosphate‐buffered saline with Tween 20
PCR: polymerase chain reaction
PLA2G4A: phospholipase A2 Group IVA
PLCB1: phospholipase C beta 1
PLCL1: phospholipase C like 1
PLD1: phospholipase D1
PVDF: polyvinylidene fluoride
RIN: RNA integrity number
RNA: ribonucleic acid
ROI: region of interest
SD: standard deviation
SDS/PAGE: sodium dodecyl sulfate–polyacrylamide gel electrophoresis
SLC: solute carrier
SRC: SRC proto‐oncogene, nonreceptor tyrosine kinase
TAC: Transcriptome Analysis Console

Previous studies have demonstrated the benefits of breastfeeding on the health and development of infants [1, 2]; these positive effects are attributed to specific components in breast milk. A systematic review of the nutritional effects on human postnatal brain development indicated that fat and energy intake are associated with increased brain volume, white matter organization, and neurodevelopment in premature infants [3]. Early‐life nutrition is an essential factor for myelination. Long‐chain fatty acids, iron, choline, sphingomyelin, and folic acid derived from diet are significantly associated with early myelination trajectories [4].

Breast milk contains major nutrients, vitamins, and minerals along with several other unique components, one of which is 1,3‐dioleoyl‐2‐palmitoylglycerol (OPO), a triacylglycerol with a palmitic acid (PA) at its sn‐2 position. OPO assists in the absorption of PA as sn‐2 palmitate, that is, 2‐palmitoyl glycerol (2‐PG), and prevents the formation of insoluble soaps that are malabsorbed. OPO also increases calcium absorption, contributes to bone health, improves stool consistency, improves fatty acid utilization, and has a positive effect on gut microbiota development [5, 6, 7, 8]. The absorbed 2‐PG is re‐esterified into triacylglycerol while the positioning of PA at the sn‐2 position is sustained and secreted re‐esterified triacylglycerol with PA at the sn‐2 position into the plasma [9]. The re‐esterified triacylglycerol with PA at the sn‐2 position can be converted to 2‐PG by lipase in the vasculature [10]. Although some proteins that transport fatty acids, for example, fatty acid binding proteins (FABPs), long‐chain acyl‐coA synthase (ACS), fatty acid transportation proteins (FATPs), fatty acid translocase (FAT/CD36), and major facilitator superfamily domain‐containing protein (Mfsd2a), have been reported [11], the mechanisms by which plasma fatty acids/glycerols cross the blood–brain barrier and enter the brain remains unclear. However, it is reasonable to investigate the effects of 2‐PG in the brain since adipose triglyceride lipase and hormone‐sensitive lipase, which hydrolyze fatty acids and generate diacylglycerols and monoacylglycerols, respectively, are expressed in the brain [12].

2‐Palmitoyl glycerol is one of the major species of brain monoacylglycerols. It is synthesized from PA containing phospholipids, and its concentration is partly controlled by the supply of its precursors to the brain [13]. Although the structure of 2‐PG is similar to that of 2‐arachidonoylglycerol (2‐AG), one of the typical endocannabinoids, it neither binds to nor activates cannabinoid receptors directly. However, 2‐PG potentiates the activity of 2‐AG [13] and this has been dubbed the ‘entourage effect’ [14]. As human breast milk is enriched in OPO, a possible hypothesis is that 2‐PG provides some advantage in the growth and brain development of infants; data on its role in the central nervous system, however, remain limited. Moreover, different lipid structures, such as PA, 2‐PG, and 3‐palmitoyl glycerol (3‐PG), may have different functions in the brain, as 1‐ and 2‐monoacylglycerol differ in their functional activity [15].

Astrocytes are the outermost part of the blood–brain barrier, a functional unit that consists of brain capillary endothelial cells, endothelial cells, pericytes, and astrocytes [16, 17]. Astrocytes play pivotal roles in brain energy metabolism, contribute to nutrient uptake from the blood, and to the synthesis of polyunsaturated fatty acids [18] and oleic acid [19]. Astrocytes also play important roles in brain development, for instance, in axonal outgrowth and in the establishment and maintenance of neuronal networks [20]. In addition, astrocytes are closely associated with neurotransmitters since they not only express neurotransmitter receptors [21] and transporters [22] but also exocytose them [23]. One major example is the glutamate/gamma aminobutyric acid (GABA)‐glutamine cycle, wherein astrocytes transport glutamate via EAAT1/EAAT2, synthesize glutamine from glutamate by glutamine synthetase, and release glutamine into the synaptic cleft [24]. GABA is generally known to be produced in the neurons; in immature brains, however, astrocytes produce GABA and release it into the extracellular space [25].

In this study, we investigated the effect of 2‐PG on astrocytes, which may potentially contribute to brain development in infants, while comparing it to PA or 3‐PG, by analyzing the gene and protein expression patterns in fetal‐derived astrocytes.

## Materials and methods

### Preparation of low glucose medium with fatty acid and monoacylglycerols

Bovine serum albumin (BSA)‐conjugated PA, 2‐PG, and 3‐PG were prepared as previously described [26]. Briefly, 500 mmol·L^−1^ of each fatty acid/monoacylglycerol was dissolved in ethanol at 55 °C. Meanwhile, 10% free fatty acid (FFA)‐free BSA was dissolved in Dulbecco's modified Eagle's medium (DMEM) containing 1.0 g·L^−1^ glucose (DMEM low glucose; NACALAI TESQUE, Kyoto, Japan) at 37 °C and subsequently filtered through Millex‐GV Syringe Filter with a 0.22 μm pore size hydrophilic polyvinylidene difluoride membrane (Merck Millipore, Burlington, USA). Each of the dissolved fatty acid/monoacylglycerols was mixed with 10% FFA‐free BSA in a 1 : 100 ratio, vortexed, and incubated at 55 °C for 15 min, followed by a 15 min conjugation in a water sonicator, until the fatty acid/monoacylglycerol‐BSA was dispersed. The vehicle control was prepared by mixing ethanol and 10% FFA‐free BSA in a 1 : 100 ratio. Fatty acid/monoacylglycerol‐BSA and the vehicle control were stored at −20 °C and melted in a water bath at 55 °C prior to use; thereafter, they were diluted in DMEM low glucose (NACALAI TESQUE) with 10% fetal bovine serum (Biowest, Nuaille, France) and 1% of Penicillin–Streptomycin‐Amphotericin B suspension (Wako, Odawara, Japan) to 100 or 120 μmol·L^−1^. The vehicle control was diluted in the same manner as the fatty acid/monoacylglycerols.

### Cell culture

Normal human fetal‐derived astrocytes (NHA; LONZA, Basel, Switzerland) from two donor lots (Lot. 0000647218 for microarray analysis and Lot. 0000672445 for other assays) were cultured in AGM Astrocyte Growth Medium Bullet Kit (LONZA) according to the manufacturer's instructions. At 80% confluence (days 5–7), cells were sub‐cultured in cell culture plates for 1 day for microarray/PCR/protein assay or in 8‐well glass chamber slides coated with poly‐l‐lysin for 6 days for immunocytochemistry, and switched to low glucose DMEM with fatty acid/monoacylglycerols. The concentrations of fatty acid/monoacylglycerols were 100 μmol·L^−1^ for microarray analysis and 120 μmol·L^−1^ for other assays. NHA were further cultured for 3 days for microarray analysis and for 2 days for other assays with daily medium changes. The difference in the concentrations of fatty acid/monoacylglycerols and culture period was an adjustment for the lot differences.

### Cell shape observation and cell proliferation assay

The cell shape was observed using bright‐field microscopy using a DMi1 inverted microscope (Leica Microsystems, Wetzlar, Germany). Cell proliferation was examined using the CellTiter 96 aqueous one‐cell proliferation assay kit (Promega, Madison, USA) as per the manufacturer's instructions. The absorbance was read with a plate reader (SpectraMax M2e, Molecular Devices, San Jose, USA) at 490 nm.

### Microarray analysis

Total RNA was extracted from three samples in each of the groups, which included the control and 2‐PG, using the RNeasy Mini Kit (Qiagen, Valencia, CA, USA) according to the manufacturer's instructions. An Agilent Bioanalyzer 2100 and RNA 6000 Nano LabChip Kit (Agilent Technologies, Palo Alto, USA) were used to determine the RNA integrity number (RIN) and RNA concentration. Three samples from the same group were mixed in equivalent amounts and used as a representative sample for each group. For transcriptome analysis, 5.5 μg fragmented, biotin‐labeled ss‐cDNA was hybridized to Clariom™ S arrays, human (Applied Biosystems; Thermo Fisher Scientific, Waltham, USA). All arrays were scanned using the Affymetrix GeneChip Command Console, which was installed on the GeneChip Scanner 3000‐7G. The Affymetrix gene chip operating system (GCOS) software was used for quality control analysis.

### Transcriptome analysis console

Array datasets were expressed as fold‐change values using the Affymetrix's transcriptome analysis console (TAC) software (version 4.0) applying the Clariom S human library for comparison with control.

### RNA extraction, 1st Strand cDNA synthesis, and quantitative PCR

Total RNA was extracted from the cell using the RNeasy Mini Kit (Qiagen) according to the manufacturer's instructions and the concentration of the extracted total RNA was measured using a spectrophotometer (Denovix DS‐11, DeNovix, Wilmington, USA). For the 1st strand cDNA synthesis, 400 ng total RNA was used with SuperScriptII Reverse Transcriptase (Invitrogen; Thermo Fisher Scientific). Briefly, 15 μL RNA solution containing 400 ng of total RNA and 1.5 μL of oligo(dT)_12–18_ primer (Invitrogen) were mixed with 1.5 μL of dNTP Mix (10 mm each) (Thermo Scientific; Thermo Fisher Scientific), denatured at 65 °C for 5 min and cooled on ice. Following the addition of 6 μL of 5X First‐Strand Buffer and 3 μL of 0.1 m dithiothreitol, the contents were gently centrifuged and 1.5 μL of SuperScript II RT was added. The contents were gently mixed and incubated at 25 °C for 10 min, at 42 °C for 50 min, and at 70 °C for 15 min for inactivation, and then cooled on ice. The 1st strand cDNA products were stored at −80 °C.

The 1st strand cDNA products were amplified using the QuantiTect SYBR Green PCR Kit (Qiagen). The following Takara Perfect Real‐Time Primer pairs were used (Takara Bio, Shiga, Japan): GAPDH (Primer set ID HA067812); SLC1A2 (Primer set ID HA270174); SLC1A3 (Primer set ID HA125772); GAD1 (Primer set ID HA270255); GAD2 (Primer set ID HA309660); SLC32A1 (Primer set ID HA171007); SLC6A1 (Primer set ID HA371268); SLC6A11 (Primer set ID HA345992); SLC6A12 (Primer set ID HA390840); SLC6A13 (Primer set ID HA332643); ABAT (Primer set ID HA121082). Real‐time quantification of RNA targets was performed with the Applied Biosystems 7500 Fast Real‐Time PCR System (Thermo Scientific). The 20 μL reaction mixture contained 2 μL of 1st strand cDNA products, 2 μL of 2X SYBR Green PCR Master Mix, and 0.5 μmol·L^−1^ of each primer. The quantitative PCR (qPCR) was performed with the following conditions: 15 min at 95 °C for holding stage; 40 cycles of 15 s at 94 °C, 30 s at 65 °C for ABAT and 62 °C for the others, and then 30 s at 72 °C. Amplification specificity was checked using the melting curve according to the manufacturer's instructions on the equipment. Normalization was performed using GAPDH as the endogenous control. Statistical analysis was performed using one‐way ANOVA, followed by the Tukey–Kramer multiple comparison test. A *P* value of <0.05 was considered statistically significant. All values are expressed as mean ± SD.

### Immunocytochemistry

Cells cultured on glass chamber slides were fixed with 4% paraformaldehyde and treated with 0.5% Triton X‐100 (MP Biomedicals, Santa Ana, USA) for 30 min at room temperature. After blocking for 1 h at room temperature using Protein Block, Serum‐Free (Dako, Carpinteria, USA), the cells were incubated with the following primary antibodies for 2 h at room temperature: rabbit anti‐SLC1A2 (1 : 200, NBP1‐20136, Novus Biologicals, Centennial, USA); rabbit anti‐GAD65/GAD67 (1 : 1000, G5163, Sigma‐Aldrich, Saint Louis, USA); mouse anti‐SLC32A1 (1 : 100, AMAB91043, Sigma‐Aldrich); rabbit anti‐SLC6A1 (1 : 200, NBP1‐59878, Novus Biologicals); mouse anti‐SLC6A11 (1 : 200, sc‐376001, Santa Cruz Biotechnology, Santa Cruz, USA); mouse anti‐SLC6A12 (1 : 200, sc‐514024, Santa Cruz Biotechnology); rabbit anti‐SLC6A13 (1 : 200, PA5‐113493, Invitrogen). For negative control, cells were incubated with antibody diluent (Dako) without primary antibodies. After washing, the cells were incubated in the corresponding Alexa 488‐labeled secondary antibodies, anti‐mouse IgG (1 : 400, 715‐545‐150, Jackson Immuno Research, West Grove, USA) or anti‐rabbit IgG (1:400, 711‐545‐152, Jackson Immuno Research), for 1 h at room temperature. After mounting using the ProLong Diamond Antifade Mountant reagent (Life Technologies, Eugene, USA), images were obtained using a laser scanning confocal microscope (FLUOVIEW FV3000, Olympus, Tokyo, Japan). The fluorescence intensity of each protein on astrocytes was quantified using ImageJ (NIH, Bethesda, MD, USA). Region of interest (ROI) of 20 × 20 pixels (square) was randomly set on the soma of astrocytes, and the average gray value within the ROI was measured by ‘Measure’ tool. One or two ROIs were set on each astrocyte, and a total of 40 ROIs from four images (10 ROIs per image) were analyzed.

### Western blot analysis

The cells cultured on a 6‐well plate were rinsed with cold phosphate‐buffered saline (PBS, Gibco; Thermo Fisher Scientific) and lysed in RIPA buffer (Cell Signaling Technology, Beverly, USA) containing a protease inhibitor cocktail (Sigma‐Aldrich), 10 mmol·L^−1^ sodium pyrophosphate (Alfa Aesar, Ward Hill, USA), 100 mmol·L^−1^ NaF (Wako), and 2 mmol·L^−1^ sodium orthovanadate (Wako). Lysates were sonicated and then centrifugation at 13 000 ***g*** for 15 min. Lysates were stored at −80 °C. Protein concentrations were measured using bicinchoninic acid protein assay kits (Takara Bio) as per the manufacturer's instructions. Protein samples (5 μg) were loaded on each lane of a sodium dodecyl sulfate‐polyacrylamide gel electrophoresis (SDS/PAGE) gel. After electrophoresis, proteins were transferred onto a polyvinylidene fluoride membrane (Bio‐Rad, Hercules, USA) and incubated with 5% skim milk in PBS‐T for 1 h. After washing, the membranes were incubated with the following primary antibodies overnight at 4 °C: rabbit anti‐GAD65/GAD67 (1 : 5000, G5163, Sigma‐Aldrich); rabbit anti‐GAPDH (1 : 10 000, GTX100118, GeneTex, Irvine, USA). After washing, the membranes were incubated with horseradish peroxidase (HRP)‐conjugated secondary anti‐rabbit antibody (1 : 10 000, Cell Signaling Technology) for 1 h at room temperature. After another round of washing, the detection was performed using Immobilon Forte Western HRP Substrate (Merck Millipore). For quantification of signal intensity of western blots, a LAS‐4000 mini image analyzer (Fujifilm, Tokyo, Japan) was used. Prestained protein markers (Bio‐Rad, Hercules, CA, USA) were used as the standard molecular mass proteins. Normalization was performed using GAPDH as the endogenous control. Statistical analysis was performed using one‐way ANOVA, followed by the Tukey–Kramer multiple comparison test. A *P* value of <0.05 was considered statistically significant. All values are expressed as mean ± SD.

### GABA ELISA

The concentration of GABA in the NHA culture supernatant was determined using the GABA ELISA Kit (ImmuSmol, Pessac, France) as per the manufacturer's instructions. The absorbance was read using a plate reader (SpectraMax M2e, Molecular Devices) at 450 nm with a 620 nm reference (A450‐620). GABA concentrations in the samples were quantified by comparing the absorbance to a reference curve prepared over a concentration range from 25 to 2500 ng·mL^−1^. Statistical analysis was performed using the Student's *t*‐test. A *P* value of <0.05 was considered statistically significant. All values are expressed as mean ± SD.

## Results

### Cell shape and cell proliferation

Exposure to PA, 2‐PG, and 3‐PG neither affected the shape of NHA cells (Fig. S1) nor their proliferation (Fig. S2).

### Genes responsive to 2‐PG in NHA

We performed transcriptome analysis in NHA cells exposed to 2‐PG (Table S1). To determine the effects potentially contributing to brain development, we focused on the pathways belonging to the nervous system and selected two groups consisting of 202 genes in total, including 113 genes associated with glutamatergic synapses (hsa04724) and 89 genes associated with GABAergic synapse (hsa04727) from the KEGG database.

A gene set differentially regulated in the 2‐PG‐exposed NHA compared with control is shown in Table 1. In the glutamatergic synapse‐related genes, we identified 15 genes (*GRIN2A*, *HOMER3*, *PLCB1*, *ITPR3*, *PLA2G4A*, *PLD1*, *ADCY8*, *GRM6*, *GRM7*, *GNG10*, *ADRBK1*, *CACNA1A(GRK3)*, *SLC1A1*, *CACNA1D*, and *SLC1A3*) showing at least a 1.5‐fold increase in expression while 6 genes (*GRIA3*, *GRIA4*, *GRIN3B*, *GRM1*, *GNGT1*, and *ADRBK2*) were suppressed. In the GABAergic synapse‐related genes, 8 genes (*GABRA5*, *GABRA6*, *SRC*, *HAP1*, *CACNA1A*, *CACNA1D*, *GNG10*, and *ADCY8*) were upregulated, while 4 genes (*GABRB2*, *PLCL1*, *CACNA1C*, and *GNGT1*) were downregulated. Out of the upregulated genes, at least 1.5‐fold increased, *SLC1A1* and *SLC1A3* are glutamate transporters, while *GABRA5* and *GABRA6* are GABA A receptors; hence, we speculated that 2‐PG may potentially change the glutamate/GABA‐glutamine cycle. Therefore, we focused on the relative expression of 15 genes, *SLC1A1*, *SLC1A2*, *SLC1A3*, *SLC1A4*, *SLC1A6*, *SLC1A7*, *GLUL*, *GAD1*, *GAD2*, *SLC32A1*, *SLC6A1*, *SLC6A11*, *SLC6A12*, *SLC6A13*, and *ABAT*, which are associated with the glutamate/GABA‐glutamine cycle, in 2‐PG‐exposed NHA. The genes with at least 1.5‐fold upregulation were only *SLC1A1* and *SLC1A3*, while *SLC32A1*, *SLC1A6*, *GAD1*, and *GAD2* were possibly upregulated since they showed more than 1.2‐fold increase (Table 2).

**Table 1:** Set of genes that were differentially regulated in 100 μmol·L^−1^ of 2‐PG‐exposed normal human fetal‐derived astrocytes compared with that in the control. The control included ethanol used to dissolve 2‐PG. *n* = 1, as a representative sample.

Criteria for selection	Number of genes^a^
Gene related to Glutamatergic synapse (KO04724, 113 genes)	
Genes upregulated in response to 2‐PG (at least 1.5‐fold)	15
Genes downregulated in response to 2‐PG (at least 1.5‐fold)	6
Genes related to GABAergic synapse (KO04727, 89 genes)	
Genes upregulated in response to 2‐PG (at least 1.5‐fold)	8
Genes downregulated in response to 2‐PG (at least 1.5‐fold)	4

^a^ Duplicate genes from different probes were excluded.

**Table 2:** Relative fold change obtained in microarray analysis of the 15 selected genes associated with GABA and glutamate in 100 μmol·L^−1^ of 2‐PG‐exposed normal human fetal‐derived astrocytes compared with those in the control. The control included ethanol used to dissolve 2‐PG. *n* = 1, as a representative sample.

Factor	Relative fold change
SLC1A3	1.85
SLC1A1	1.85
SLC32A1	1.39
SLC1A6	1.26
GAD2	1.24
GAD1	1.20
SLC6A1	1.12
SLC6A11	1.11
ABAT	1.10
SLC6A12	1.03
SLC1A4	0.84
SLC1A2	0.80
GLUL	0.80
SLC1A7	0.74
SLC6A13	0.74

### Validation of GABA‐related gene expression

**Fig. 1:**
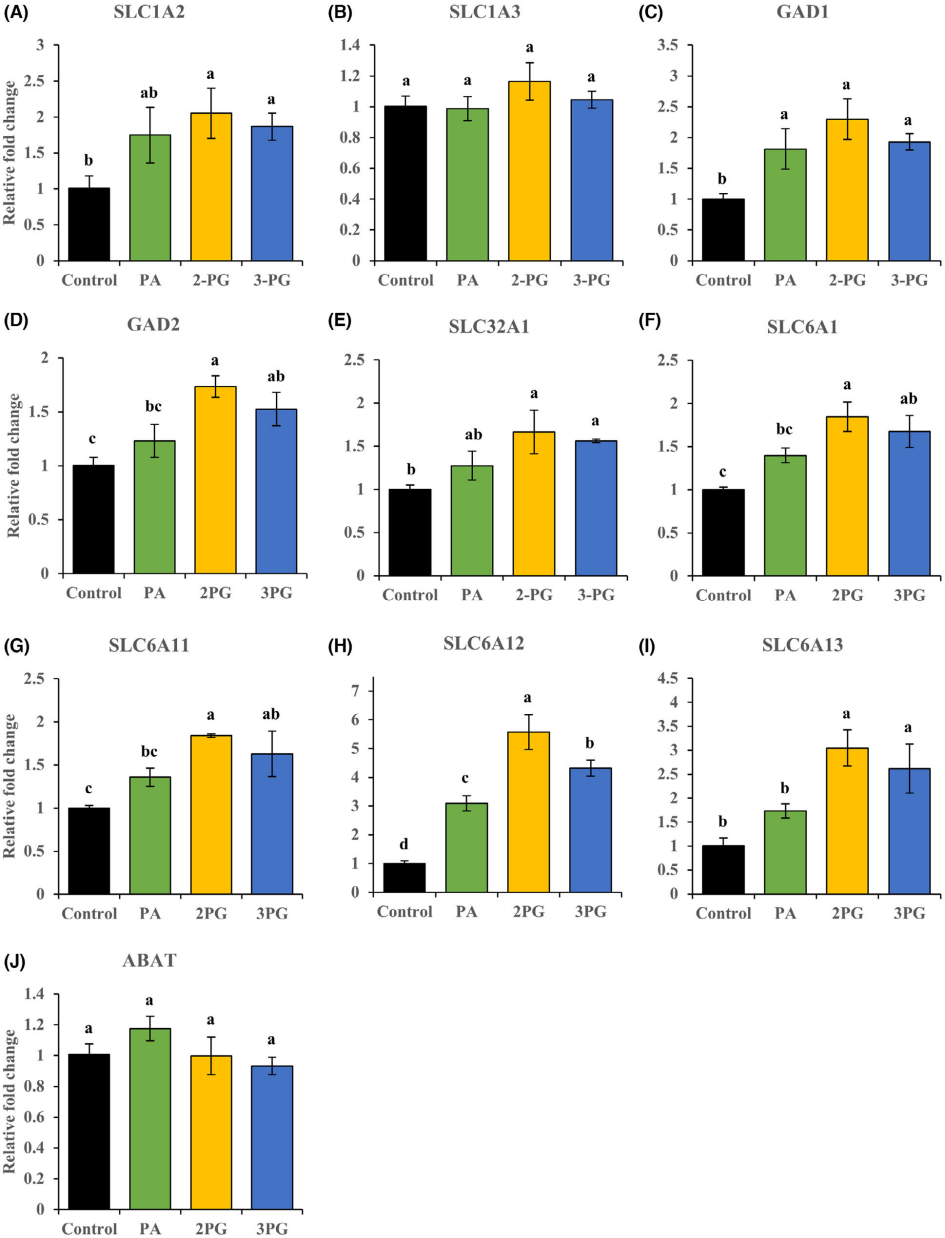
Relative fold change in mRNA expression in control, PA‐, 2‐PG‐, and 3‐PG‐exposed normal human fetal‐derived astrocytes using qPCR. The 10 selected genes associated with glutamate/GABA‐glutamine cycle in control, PA‐, 2‐PG‐, and 3‐PG‐exposed normal human fetal‐derived astrocytes were determined using qPCR analysis. The concentration of fatty acid/monoacylglycerols was 120 μmol·L^−1^. The control included ethanol used to dissolve fatty acid/monoacylglycerols. All data were normalized to GAPDH expression and expressed relative to the control: *n* = 3; mean ± SD; Data were analyzed using one‐way ANOVA followed by the Tukey–Kramer multiple comparison test. Significant difference (*P* < 0.05) was observed between the values marked with different characters. *SLC1A2* (A), *SLC1A3* (B), *GAD1* (C), *GAD2* (D), *SLC32A1* (E), *SLC6A1* (F), *SLC6A11* (G), *SLC6A12* (H), *SLC6A13* (I), and *ABAT* (J).

Among the 15 genes related to glutamate/GABA‐glutamine cycle, we selected 10 namely *SLC1A2*, *SLC1A3*, *GAD1*, *GAD2*, *SLC32A1*, *SLC6A1*, *SLC6A11*, *SLC6A12*, *SLC6A13*, and *ABAT*, focusing on GABAergic genes, most of which are dominantly expressed in immature astrocytes. We also investigated the expression of these genes in PA‐ or 3‐PG‐exposed NHA to compare with that in 2‐PG‐exposed NHA. We validated these gene expression changes using qPCR and found that all the three fatty acid/monoacylglycerols tested, namely, PA, 2‐PG, and 3‐PG, significantly upregulated *GAD1*, *SLC6A1*, and *SLC6A12* compared with that in the control (*P* < 0.05) (Fig. 1C,F,H). Monoacylglycerols, 2‐PG and 3‐PG, significantly upregulated *SLC1A2*, *GAD2*, *SLC32A1*, *SLC6A11*, and *SLC6A13* compared with that in the control (*P* < 0.05), while no significant differences in response to PA (Fig. 1A,D,E,G,I) were observed. Moreover, 2‐PG significantly increased the expression of *GAD2*, *SLC6A1*, *SLC6A11*, *SLC6A12*, and *SLC6A13* compared with PA (*P* < 0.05) (Fig. 1D,F–I). None of the fatty acid/monoacylglycerols affected the gene expression of *SLC1A3* and *ABAT* (Fig. 1B,J).

### Protein expression of differentially expressed genes

**Fig. 2:**
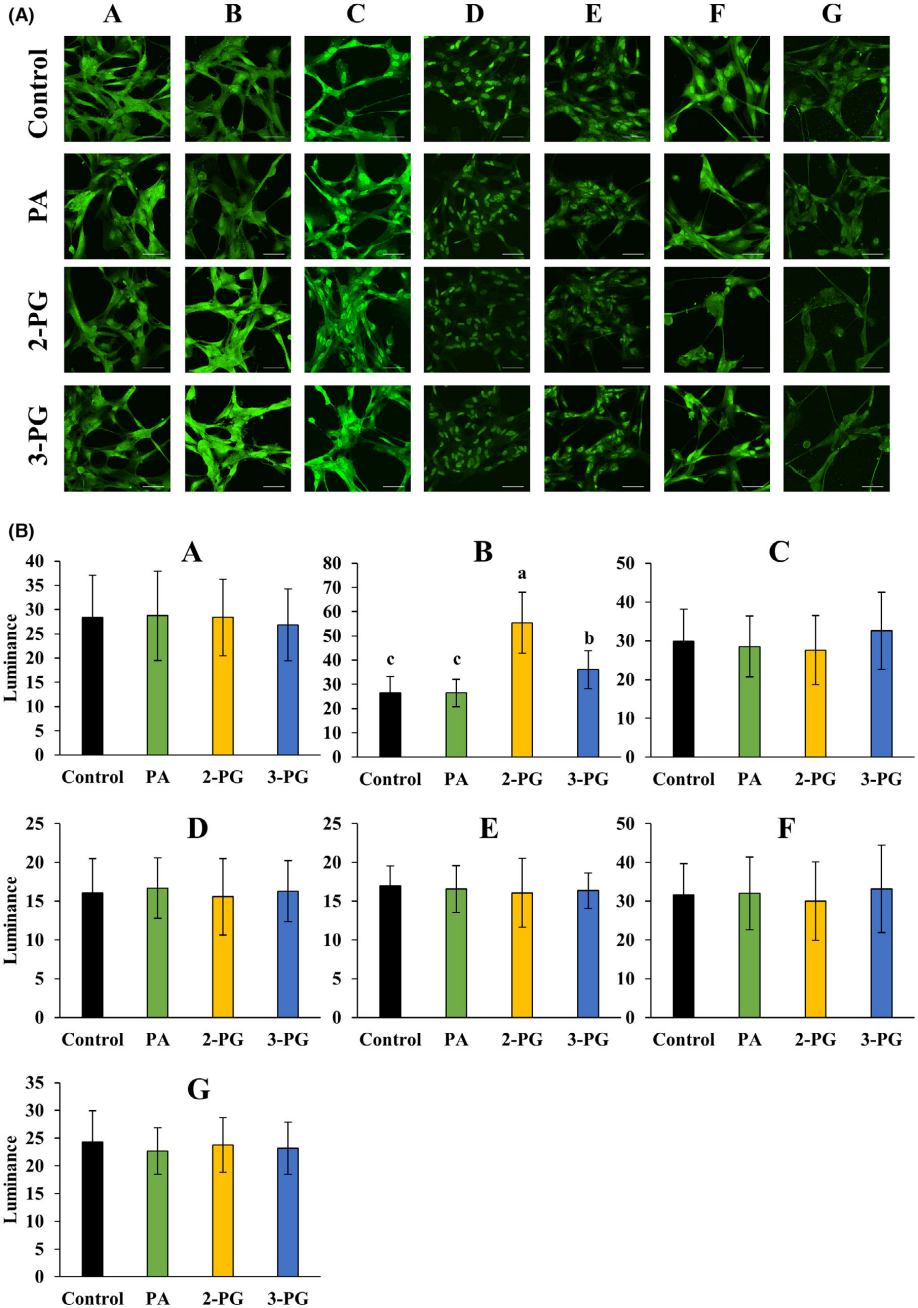
Immunocytochemistry in control, PA‐, 2‐PG‐, and 3‐PG‐exposed normal human astrocytes. (A) Images of protein staining (scale bar = 50 μm). The target proteins are shown in green. (B) Quantitative values of protein staining: *n* = 30 ~ 40; mean ± SD; Data were analyzed using one‐way ANOVA followed by the Tukey–Kramer multiple comparison test. Significant difference (*P* < 0.05) was observed between the values marked with different characters. The concentration of fatty acid/monoacylglycerols was 120 μmol·L^−1^. The control included ethanol used to dissolve fatty acid/monoacylglycerols. SLC1A2 (a), GAD1/GAD2 (b), SLC32A1 (c), SLC6A1 (d), SLC6A11 (e), SLC6A12 (f), and SLC6A13 (g).

To investigate whether the protein expression patterns corroborated with the differences in mRNA levels, we performed an immunocytochemistry analysis of the proteins encoded by genes (*SLC1A2*, *GAD1*, *GAD2*, *SLC32A1*, *SLC6A1*, *SLC6A11*, *SLC6A12*, *SLC6A13*) that were modulated upon exposure to PA, 2‐PG, or 3‐PG. GAD1 and GAD2 were detected together due to the primary antibody specification. We confirmed the expression of all these proteins in the control, PA‐, 2‐PG‐, and 3‐PG‐exposed NHA (Fig. 2A). The luminance of GAD1/2 was significantly higher in 2‐PG‐ and 3‐PG‐exposed cells compared with that in the control, furthermore, it was also significantly higher in 2‐PG‐exposed cells than the one in PA‐exposed cells (Fig. 2B‐b). No differences were seen for other proteins (Fig. 2B).

### Validation of GAD1/2 protein expression

**Fig. 3:**
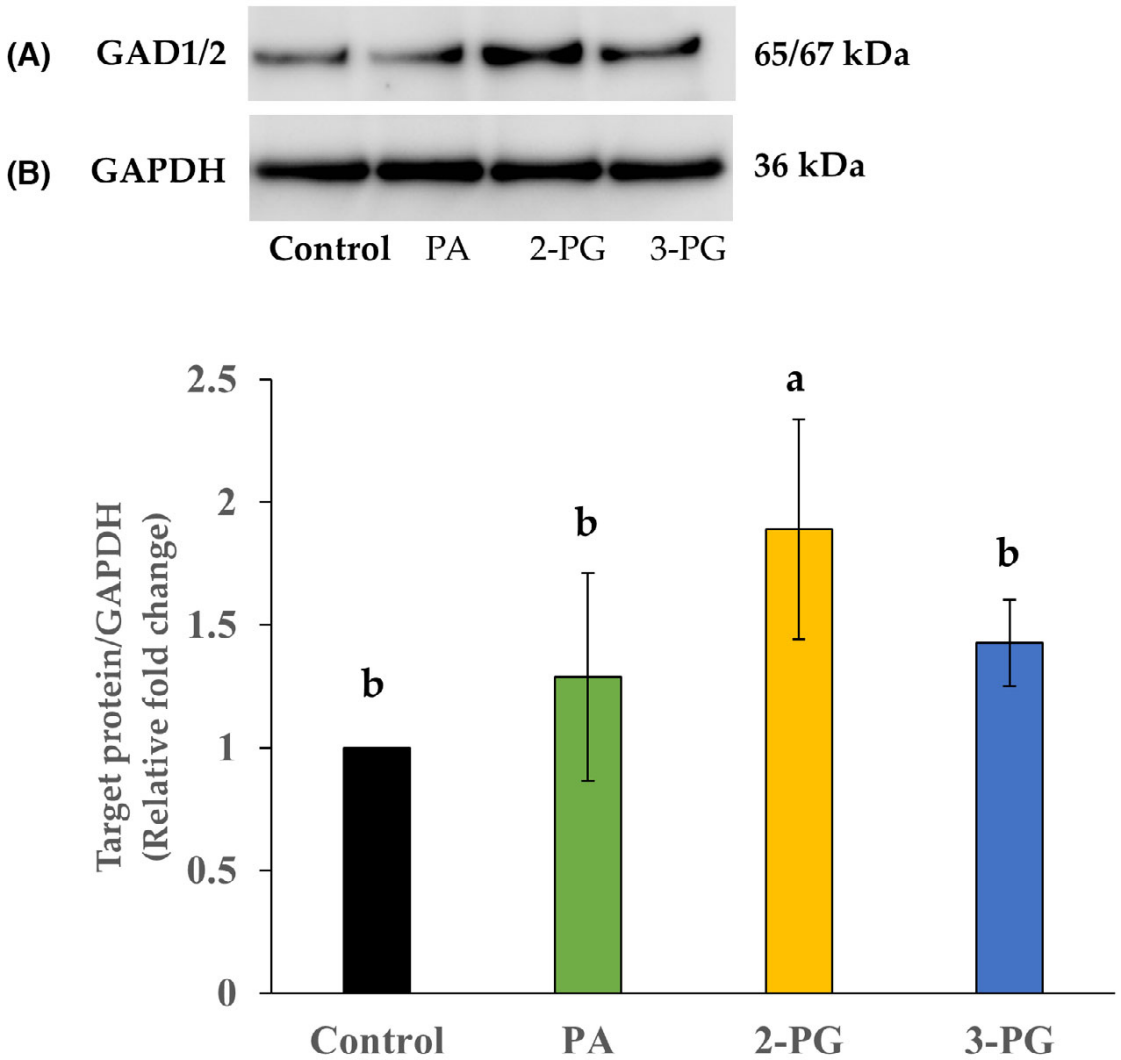
Western blot analysis of GAD1/2 expression in control, PA‐, 2‐PG‐, and 3‐PG‐exposed normal human astrocytes. The concentration of fatty acid/monoacylglycerols was 120 μmol·L^−1^. The control included ethanol used to dissolve fatty acid/monoacylglycerols. (A) Representative immunoblots of GAD1/2 and GAPDH; (B) average fold change of GAD1/2 with respect to the housekeeping gene GAPDH using densitometric analysis: *n* = 3; mean ± SD; Data were analyzed using one‐way ANOVA followed by the Tukey–Kramer multiple comparison test. Significant difference (*P* < 0.05) was observed between the values marked with different characters.

To validate the protein expression of GAD1/2 that was differentially stained between groups by immunocytochemistry, we performed western blot analysis. We detected the 65/67 kDa protein in the control, and PA‐, 2‐PG‐, and 3‐PG‐exposed NHA. The relative fold change was significantly increased in 2‐PG‐exposed cells compared with that in the control (*P* < 0.05; Fig. 3). There was no significant difference between other groups.

### GABA concentration in 2‐PG‐exposed NHA supernatant

**Fig. 4:**
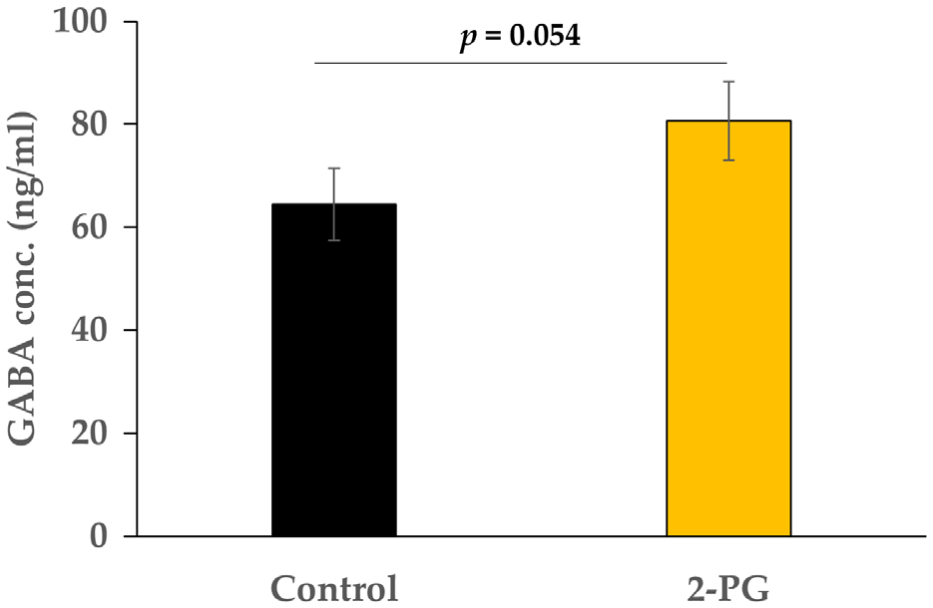
GABA concentration in the culture supernatant of the control and 2‐PG‐exposed normal human astrocytes. The concentration of fatty acid/monoacylglycerols was 120 μmol·L^−1^. The control included ethanol used to dissolve 2‐PG: *n* = 3; mean ± SD; Data were analyzed using Student's *t*‐test.

To investigate whether the concentration of GABA in culture supernatant was affected following the altered expression of GAD1/2 protein, we determined the concentration GABA in the control and 2‐PG‐exposed NHA supernatant using ELISA. Although statistical significance was not found, the concentration of GABA in 2‐PG‐exposed NHA tended to be higher than that in the control (Fig. 4).

**Fig. 5:**
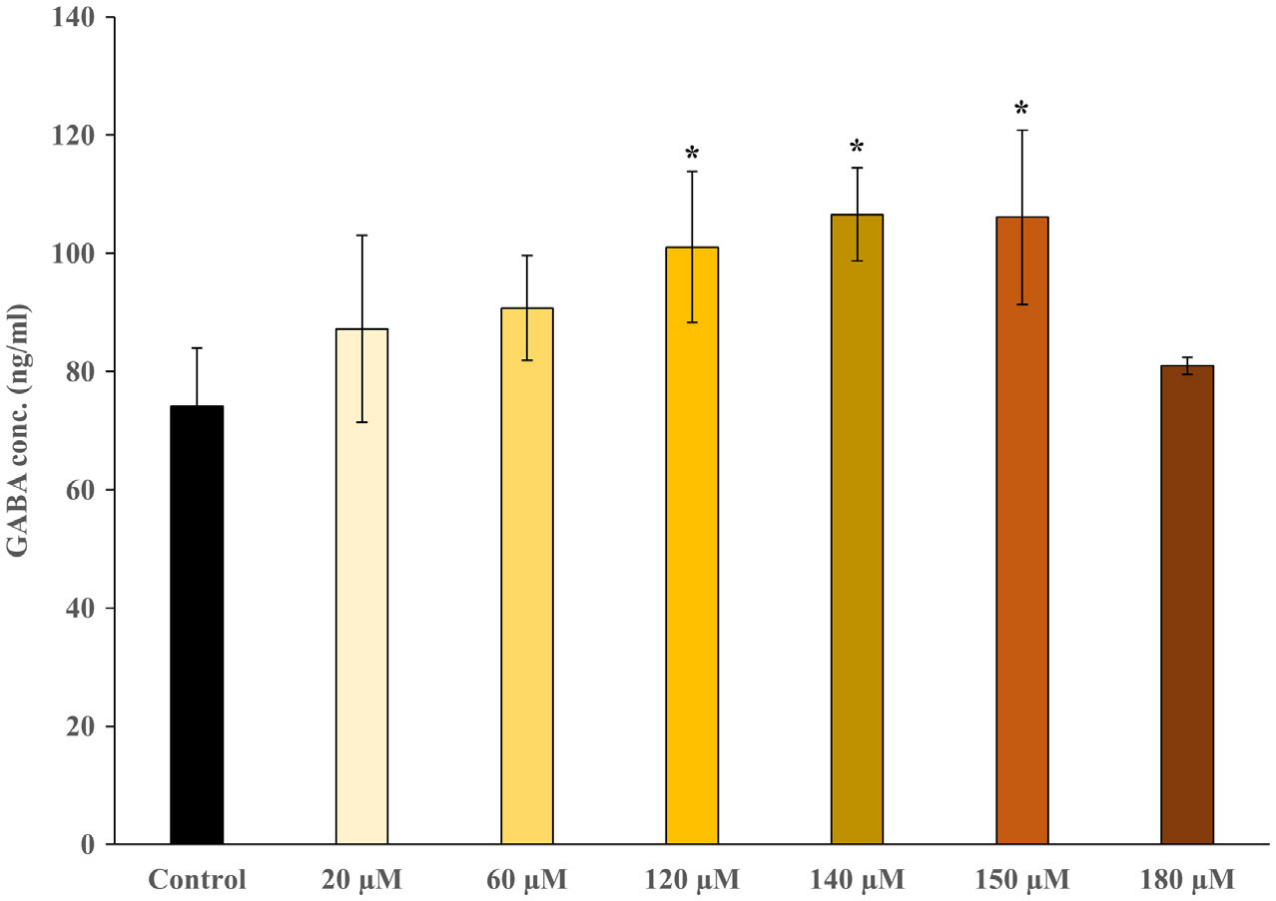
Dose‐dependence effect of 2‐PG on GABA production. The concentrations of 2‐PG were 20, 60, 120, 140, 150, and 180 μmol·L^−1^ (indicated ‘μm’ in the figure). The control included ethanol used to dissolve 2‐PG: *n* = 3; mean ± SD; Data were analyzed using one‐way ANOVA followed by the Dunnett's test. *P < 0.05 indicates a significant difference.

**Fig. 6:**
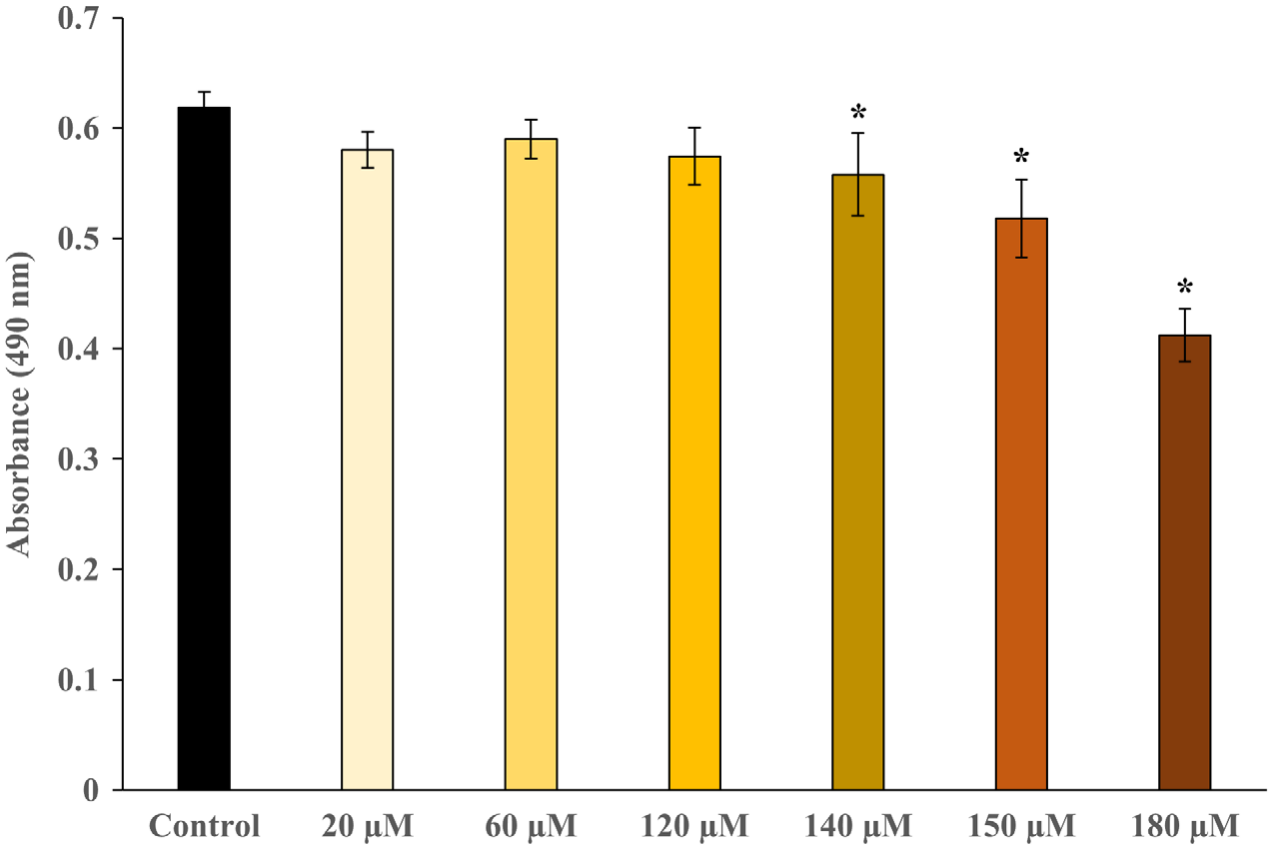
Dose‐dependent effect of 2‐PG on cell proliferation. The concentrations of 2‐PG were 20, 60, 120, 140, 150, and 180 μmol·L^−1^ (indicated ‘μm’ in the figure). The control included ethanol used to dissolve 2‐PG: *n* = 3; mean ± SD; Data were analyzed using one‐way ANOVA followed by the Dunnett's test. *P < 0.05 indicates a significant difference.

Additionally, we examined the dose‐dependent effect of 2‐PG exposure on GABA concentration and found that GABA concentration increased 2‐PG concentration‐dependent and plateaued at 140 μmoL·L^−1^ of 2‐PG and then decreased (Fig. 5). Significant increases in GABA concentrations were observed at 2‐PG concentrations of 120, 140, and 150 μmoL·L^−1^ compared to the control, whereas GABA concentration at 180 μmoL·L^−1^ of 2‐PG returned to the same level with the control. In the cell proliferation assay, significant decreases in cell viability were observed at 140, 150, and 180 μmoL·L^−1^ of 2‐PG (Fig. 6).

## Discussion

In this study, treatment with 2‐PG altered the mRNA and protein expression of GAD in NHA and also promoted GABA synthesis.

2‐Palmitoyl glycerol significantly upregulated the expression of *GAD1* and *GAD2* mRNAs but did not alter that of *ABAT* mRNA (encoding 4‐aminobutyrate aminotransferase, which is responsible for the catabolism of GABA) in astrocytes. The protein expression of GAD1/2 was also upregulated by 2‐PG. Furthermore, the concentration of GABA in culture supernatant of 2‐PG‐exposed NHA increased 2‐PG concentration‐dependent and significantly increased at 120, 140, and 150 μmoL·L^−1^ of 2‐PG. These changes at least up to 120 μmoL·L^−1^ of 2‐PG were not due to the effect of 2‐PG on the survival or death of astrocytes, because the 2‐PG treatment did not change the cell shape and proliferation. While, the decrease in GABA concentration at 180 μmoL·L^−1^, high‐concentration of 2‐PG, is likely due to decreasing cell viability. Glutamate can be a substrate not only for glutamine but also for GABA, a major inhibitory neurotransmitter. GABA contributes to neural development in the developing brain by controlling chemokinesis [27] of neurons and synaptic plasticity [28]. In immature brain, astrocytes produce and release GABA into the extracellular space [25]. The level of GAD1, which synthesizes GABA from glutamate, decreases in astrocytes as GABA neurons develop [29]. Our results suggest that 2‐PG may contribute to neural development by promoting GABA synthesis from astrocytes during brain development.

Although the changes in protein expression following the upregulation of mRNAs were not detected except for GAD, which may be because of inadequate protein detection sensitivity, we describe the possibilities for the effect of 2‐PG on astrocytes from the perspective of the mRNA changes.

>2‐Palmitoyl glycerol significantly upregulated *SLC1A2* mRNA expression in NHA. To remove glutamate from the synaptic cleft by astrocytes, it is important to control the balance between excitation and inhibition. Immature astrocytes rely on the subtype EAAT1 (encoded by *SLC1A3*) for glutamate uptake, and glutamate uptake increases as the subtype EAAT2 (encoded by *SLC1A2*) increases during their functional maturation [30]. Our results suggest that 2‐PG might promote glutamate uptake. Incidentally, 2‐PG upregulated *SLC1A2*, but not *SLC1A3*, suggesting that it could potentially promote astrocyte maturation.

The mRNA expression of all subtypes of GABA transporters, including GAT1 (encoded by *SLC6A1*), GAT2 (encoded by *SLC6A13*), GAT3 (encoded by *SLC6A11*), and BGT1 (encoded by *SLC6A12*), were significantly upregulated by 2‐PG. A large impact was observed particularly on SLC6A13, which is specifically expressed in the neonatal brain, and on SLC6A12, which is only sparsely expressed [31]. GABA acts as an excitatory transmitter during brain development [32]. Controlling extracellular GABA levels via GABA transporters is an important function of astrocytes [33]. Therefore, 2‐PG might also facilitate astrocytes' removal of excess GABA from the synaptic cleft.

The effects of 3‐PG on mRNA expressions related to GABAergic genes were similar to those of 2‐PG, while the effects of PA were limited compared to those of 2‐PG and 3‐PG. This suggests that the structure of monoacylglycerols, especially 2‐PG, may be more important for the activation of GABAergic genes than that of FFA in immature brain, considering breast milk contains high OPO and the sn‐2 position of OPO is sustained in the plasma [9].

We used TAC analysis to show that 2‐PG may alter glutamate and GABA signaling since 2‐PG altered the gene expression of GABA receptors, glutamate receptors, and the subunits of the calcium voltage‐gated channel, CACNA.

This study has several limitations. First, the use of a single representative sample for the microarray possibly contributed to the conflicting results between the microarray and qPCR: microarray showed SLC1A3 upregulation, while qPCR showed no changes; microarray showed SLC1A2 and SLC6A13 downregulation, while qPCR showed upregulation. Second, although the mRNA expression of GAT1, GAT2, GAT3, and BGT1 was significantly upregulated by 2‐PG, these changes were not confirmed at the protein level, possibly due to the effect of 2‐PG on posttranscriptional regulation and protein degradation. Third, there was no significant increase in the extracellular level of GABA after 2‐PG treatment. This may be attributed to an increased uptake of GABA by the mRNA upregulation of GABA transporters, although such upregulation was not confirmed at the protein level.

## Conclusions

This study indicates that 2‐PG contributes to the promotion of GABA synthesis by astrocytes. Considering the transient expression of GAD1 in astrocytes during brain development, the role of 2‐PG in promoting GABA synthesis may be specific to the developing, rather than the mature brain. To the best of our knowledge, this is the first study to observe that 2‐PG promotes GABA synthesis via its own effect but not through the entourage effect of 2‐AG. Further research is warranted to determine the function of monoacylglycerols, like 2‐PG, and FFAs in the brain. Since the experiments were performed under *in vitro* conditions, the collaborative effects in the context of the other cell types and tissue environment remain to be determined. These results should be validated via *in vivo* experiments in other models to further investigate the effects on neurogenesis and other aspects of neural development. Future studies should also investigate the effects of other monoacylglycerols and FFAs on early neural development.

## Conflict of interest

M. Tsuboi and Y. Nakamura are employed by Meiji Co., Ltd., Tokyo. H. Sakuma received a research fund from Meiji Co., Ltd.

### Peer Review

The peer review history for this article is available at https://www.webofscience.com/api/gateway/wos/peer‐review/10.1002/2211‐5463.13649.

## Author contributions

MT involved in conceptualization; MT and HS involved in methodology; MT and HS involved in software; MT and HS involved in validation; MT involved in formal analysis; MT and HS involved in investigation; MT and HS involved in resources; MT and HS involved in data curation; MT involved in writing—original draft preparation; HS and YN involved in writing—review and editing; MT and HS involved in visualization; HS and YN involved in supervision; MT involved in project administration. All authors have read and agreed to the published version of the manuscript.

## Supporting information


**Figure S1.** Bright‐field images of pre‐exposed/control, PA‐, 2‐PG‐, and 3‐PG‐exposed normal human fetal‐derived astrocytes (NHAs).

**Figure S2.** Cell proliferation assay was performed using control, PA‐, 2‐PG‐, and 3‐PG‐exposed normal human fetal‐derived astrocytes using the MTS assay.

**Table S1.** The results of microarray for genes that altered more than 1.5‐fold change and housekeeping genes of 2‐PG‐exposed normal human fetal‐derived astrocytes compared with those in the control.

## Data Availability

The datasets analyzed during the current study are available from the corresponding author upon reasonable request.
