# Supplementary figures and images for: Direct effect of 2‐palmitoyl glycerol on promotion of gamma aminobutyric acid synthesis in normal human fetal‐derived astrocytes
Source: FEBS Open Bio. 2023 May 24;13(7):1320–32. doi: 10.1002/2211-5463.13649 (PMC10315726; doi:10.1002/2211-5463.13649)

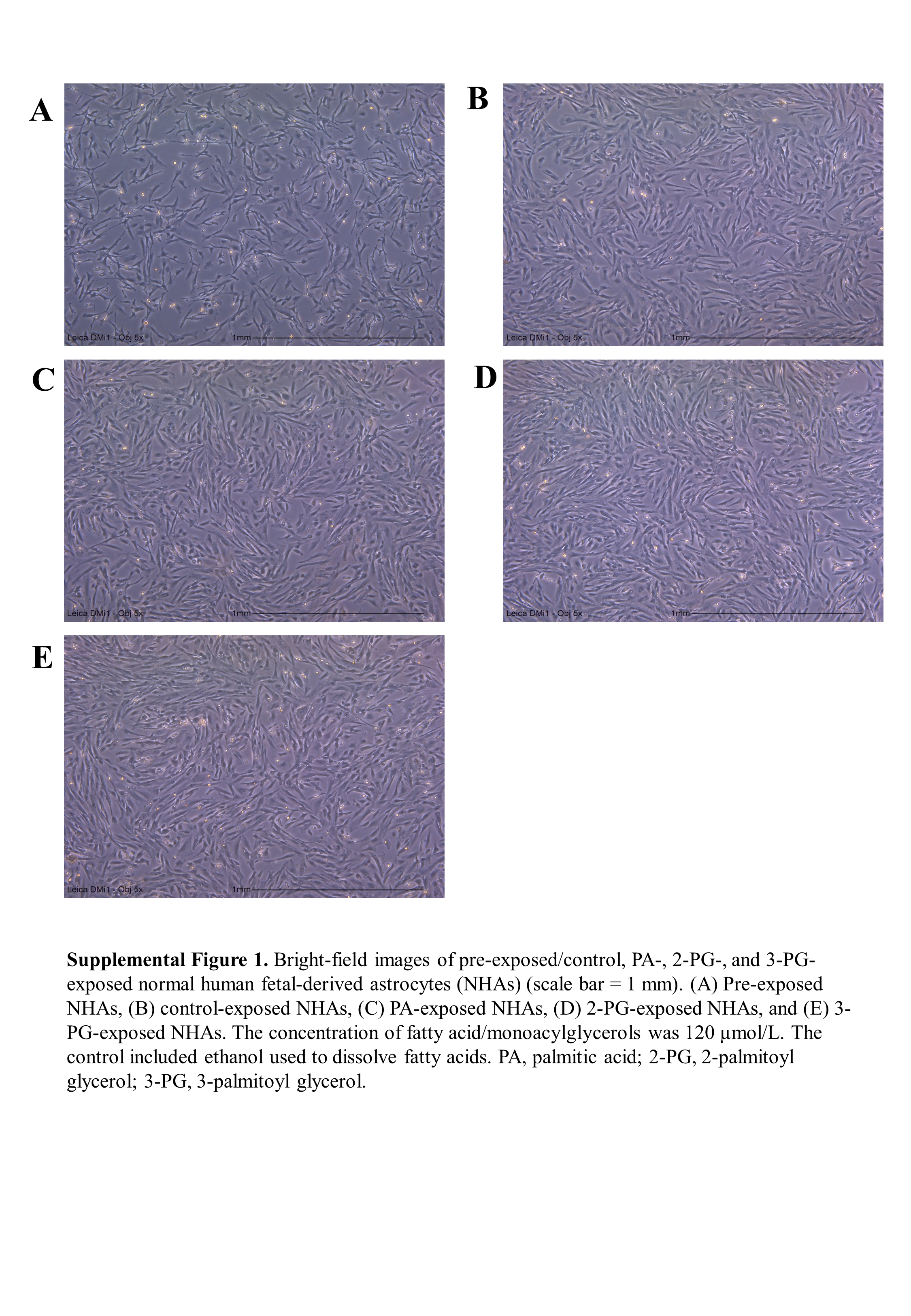

Supplement: Supplementary file 1 — Figure S1. Bright‐field images of pre‐exposed/control, PA‐, 2‐PG‐, and 3‐PG‐exposed normal human fetal‐derived astrocytes (NHAs). [file FEB4-13-1320-s003.tif]

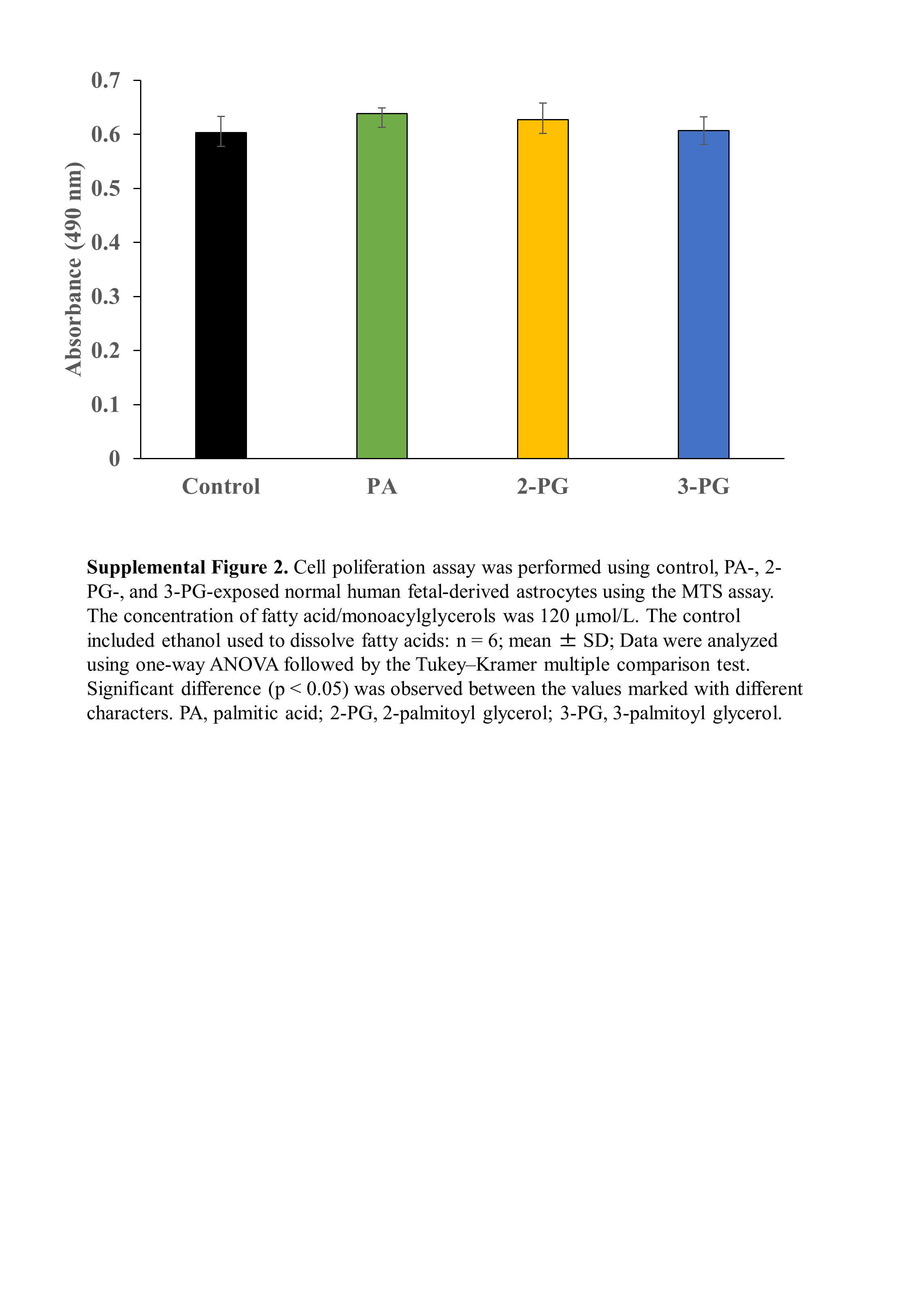

Supplement: Supplementary file 2 — Figure S2. Cell proliferation assay was performed using control, PA‐, 2‐PG‐, and 3‐PG‐exposed normal human fetal‐derived astrocytes using the MTS assay. [file FEB4-13-1320-s001.tif]
