# Supplementary material for: Direct effect of 2‐palmitoyl glycerol on promotion of gamma aminobutyric acid synthesis in normal human fetal‐derived astrocytes
Source: FEBS Open Bio. 2023 May 24;13(7):1320–32. doi: 10.1002/2211-5463.13649 (PMC10315726; doi:10.1002/2211-5463.13649)
Supplement: Supplementary file 3 — Table S1. The results of microarray for genes that altered more than 1.5‐fold change and housekeeping genes of 2‐PG‐exposed normal human fetal‐derived astrocytes compared with those in the control. [file FEB4-13-1320-s002.docx]

| Factor | Relative Fold Change |  | Factor | Relative Fold Change |  | Factor | Relative Fold Change |  | Factor | Relative Fold Change |
| --- | --- | --- | --- | --- | --- | --- | --- | --- | --- | --- |
| MMP1 | 15.45 |  | USP17L11; | 3.56 |  | NPNT | 2.91 |  | GREM1 | 2.62 |
| HIST1H2BM | 12.91 |  | USP17L18; |  |  | USP17L26; | 2.91 |  | FOSL1 | 2.60 |
| HAS2 | 7.84 |  | USP17L20 |  |  | USP17L24 |  |  | AKR1B15 | 2.60 |
| RRM2 | 7.26 |  | USP17L17 | 3.56 |  | PLAUR | 2.91 |  | TOR4A | 2.60 |
| CDC6 | 6.63 |  | flawmawbo; | 3.51 |  | IL1B | 2.91 |  | BUB1B | 2.60 |
| IL6 | 6.59 |  | GBP3 |  |  | ANLN | 2.91 |  | SMC6 | 2.58 |
| SPP1 | 6.11 |  | NCAPG | 3.48 |  | POGZ | 2.89 |  | ZKSCAN7 | 2.58 |
| NT5E | 5.86 |  | DDIT3 | 3.48 |  | OCLN | 2.89 |  | SGOL1 | 2.58 |
| USP17L15 | 5.50 |  | FAM83D | 3.46 |  | AOX1 | 2.89 |  | DUSP6 | 2.57 |
| HIST1H1B | 5.24 |  | CENPF | 3.41 |  | CEP55 | 2.87 |  | NRIP3 | 2.57 |
| HIST1H2AG | 5.21 |  | PARM1 | 3.41 |  | SHC3 | 2.85 |  | FAM43A | 2.57 |
| NDC80 | 5.21 |  | ITGA2 | 3.39 |  | ATAD5 | 2.85 |  | NELL2 | 2.57 |
| CDCP1 | 4.86 |  | GRIN2A | 3.36 |  | ADGRL4 | 2.83 |  | MYBL2 | 2.57 |
| ATF3 | 4.82 |  | CLDN11 | 3.34 |  | HMMR | 2.83 |  | SEMA7A | 2.57 |
| PBK | 4.79 |  | HYLS1 | 3.34 |  | PPAN-P2RY11; | 2.83 |  | PRC1 | 2.57 |
| TOP2A | 4.69 |  | FABP3 | 3.32 |  | P2RY11; |  |  | AXL | 2.57 |
| MARCH4 | 4.56 |  | KIAA1549L | 3.32 |  | PPAN |  |  | RAD21L1 | 2.57 |
| USP17L28; | 4.50 |  | CDC34 | 3.32 |  | SKP2 | 2.83 |  | FKBPL | 2.57 |
| USP17L5; |  |  | SLC14A1 | 3.32 |  | HJURP | 2.83 |  | MKI67 | 2.57 |
| USP17L24 |  |  | LOC105377348; | 3.32 |  | MCTP1 | 2.81 |  | TMEM200A | 2.55 |
| AP1S1 | 4.23 |  | RP11-10L12.4; |  |  | C14orf39 | 2.81 |  | TTC32 | 2.55 |
| KIF14 | 4.17 |  | UBE2D3; |  |  | CXCL8 | 2.79 |  | CCND1 | 2.55 |
| IRAK2 | 4.03 |  | pugo |  |  | ANPEP | 2.79 |  | CD274 | 2.53 |
| USP17L23 | 4.03 |  | TPX2 | 3.27 |  | DEPDC1 | 2.79 |  | SLC9A7 | 2.53 |
| KIF20A | 4.03 |  | PLK1 | 3.25 |  | FANCI | 2.79 |  | KCNJ2 | 2.53 |
| USP17L27; | 3.97 |  | HMOX1 | 3.25 |  | CCDC65 | 2.79 |  | HMG20A | 2.53 |
| USP17L28 |  |  | PLAT | 3.25 |  | MICU3 | 2.79 |  | TSLP | 2.53 |
| USP17L28; | 3.97 |  | GPNMB | 3.23 |  | GJC1 | 2.79 |  | HIST1H3B | 2.51 |
| USP17L30 |  |  | USP17L18; | 3.16 |  | HMGA1 | 2.77 |  | SDR42E1 | 2.51 |
| USP17L29; | 3.97 |  | USP17L11 |  |  | TOX2 | 2.77 |  | SPRED3 | 2.51 |
| USP17L5 |  |  | PPP6R1 | 3.16 |  | FLI1 | 2.75 |  | MARCH3 | 2.50 |
| USP17L25; | 3.97 |  | FOXM1 | 3.14 |  | PREX1 | 2.75 |  | NCAPH | 2.50 |
| USP17L30 |  |  | USP17L24; | 3.12 |  | CLEC2B | 2.75 |  | ZNF512 | 2.50 |
| NOG | 3.94 |  | USP17L26; |  |  | L3MBTL4 | 2.75 |  | TMEM174 | 2.50 |
| SKA1 | 3.89 |  | USP17L5; |  |  | OR5K2 | 2.73 |  | H2AFX | 2.50 |
| MLPH | 3.81 |  | USP17L27; |  |  | PAK3 | 2.73 |  | TRHDE | 2.50 |
| EZH2 | 3.81 |  | USP17L29; |  |  | DKK1 | 2.71 |  | GRAMD3 | 2.50 |
| FAM111B | 3.78 |  | USP17L30 |  |  | PLAU | 2.71 |  | MAFK | 2.48 |
| KIF4A | 3.78 |  | USP17L25; | 3.12 |  | CCNB2 | 2.69 |  | DGKI | 2.48 |
| CDCA2 | 3.76 |  | USP17L27 |  |  | SERPINB7 | 2.69 |  | WRB | 2.48 |
| KIAA0101; | 3.76 |  | ITPR3 | 3.12 |  | HHIP | 2.66 |  | TTK | 2.48 |
| CSNK1G1 |  |  | ABCA1 | 3.10 |  | MTFR1 | 2.66 |  | C4orf26 | 2.46 |
| VEPH1 | 3.76 |  | SHCBP1 | 3.05 |  | UBASH3B | 2.66 |  | AGTR1 | 2.46 |
| DNER | 3.71 |  | CRY2 | 3.03 |  | TTC5 | 2.66 |  | CLGN | 2.46 |
| SHISA2 | 3.68 |  | HIST1H3G | 3.01 |  | HIST1H2AB | 2.66 |  | EDRF1 | 2.45 |
| USP17L22; | 3.66 |  | PDE10A | 3.01 |  | KIRREL3 | 2.64 |  | ASPM | 2.45 |
| USP17L20 |  |  | NOV | 3.01 |  | MICALCL | 2.64 |  | CDC20 | 2.45 |
| FBXO5 | 3.63 |  | HSP90B1 | 3.01 |  | TAF1A | 2.64 |  | TRAT1 | 2.45 |
| TXNIP | 3.63 |  | FRMD6 | 2.97 |  | TRIM55 | 2.64 |  | AK5 | 2.45 |
| EXO1 | 3.61 |  | TGFBR3 | 2.95 |  | LINC01587 | 2.62 |  | OR11H1 | 2.43 |
| USP17L13 | 3.58 |  | DLGAP5 | 2.93 |  | RCL1 | 2.62 |  | USP17L21 | 2.43 |
| TEK | 3.58 |  | KIF15 | 2.93 |  | PLK4 | 2.62 |  | MED14OS | 2.43 |
| CEP128 | 3.56 |  | PTX3 | 2.93 |  | BRCA2 | 2.62 |  | LCP1 | 2.43 |

Supplemental Table 1. The results of microarray for genes that altered more than 1.5-fold change and housekeeping genes of 2-PG-exposed normal human fetal-derived astrocytes compared with those in the control. The control included ethanol used to dissolve 2-PG.

| Factor | Relative Fold Change |  | Factor | Relative Fold Change |  | Factor | Relative Fold Change |  | Factor | Relative Fold Change |
| --- | --- | --- | --- | --- | --- | --- | --- | --- | --- | --- |
| ZNF546 | 2.43 |  | ZKSCAN7 | 2.25 |  | OR5J2 | 2.14 |  | BAHD1 | 2.07 |
| SMAGP | 2.43 |  | CD19 | 2.25 |  | NRG4 | 2.14 |  | CEP97 | 2.07 |
| SKA3 | 2.43 |  | MT1M | 2.25 |  | SPX | 2.14 |  | RRS1 | 2.07 |
| MAP3K6 | 2.43 |  | CYB561 | 2.25 |  | MMP9 | 2.14 |  | ARPC5L | 2.07 |
| PLA2G4A | 2.43 |  | SOGA1 | 2.25 |  | FSD1L | 2.14 |  | CD82 | 2.07 |
| CDH4 | 2.41 |  | EMP1 | 2.23 |  | ADAM28 | 2.13 |  | PKD1L2 | 2.07 |
| HIST2H4B; | 2.41 |  | OR56A4 | 2.23 |  | C17orf47 | 2.13 |  | DUSP1 | 2.06 |
| HIST2H4A |  |  | DTL | 2.23 |  | MEX3B | 2.13 |  | FGF5 | 2.06 |
| MAATS1 | 2.39 |  | CCNA2 | 2.23 |  | NUDC | 2.13 |  | HIST1H2AJ | 2.06 |
| FGF16 | 2.39 |  | HIST1H2AM; | 2.23 |  | KIF15 | 2.13 |  | NUP205 | 2.06 |
| PTGS2 | 2.39 |  | HIST1H3J |  |  | PRR35 | 2.13 |  | HKDC1 | 2.06 |
| XRCC2 | 2.39 |  | CNTN1 | 2.23 |  | AHCY | 2.13 |  | SGMS2 | 2.06 |
| TPBG | 2.38 |  | CFAP54 | 2.23 |  | PPP1R15A | 2.13 |  | CSN1S1 | 2.06 |
| ANXA10 | 2.38 |  | SERTAD3 | 2.23 |  | SRC | 2.13 |  | PGAM4 | 2.06 |
| IFNL3 | 2.38 |  | SRXN1 | 2.23 |  | ZIC3 | 2.13 |  | GEMIN4 | 2.06 |
| TAS2R19 | 2.38 |  | OPRL1 | 2.23 |  | LIMD1 | 2.11 |  | LPXN | 2.06 |
| TBXAS1 | 2.38 |  | USP17L22 | 2.22 |  | FAM65B | 2.11 |  | KCNIP3 | 2.06 |
| CHST11 | 2.38 |  | EVI5 | 2.22 |  | OSGIN1 | 2.11 |  | ARL4C | 2.04 |
| TMEM156 | 2.36 |  | PSMC6 | 2.22 |  | RP1-122P22.2; | 2.11 |  | LRRC8C | 2.04 |
| SORBS3 | 2.36 |  | TP53TG3B | 2.22 |  | RIN2 |  |  | LMNB1 | 2.04 |
| SEC11C | 2.36 |  | ERO1B | 2.22 |  | CSAG3 | 2.11 |  | DNAH5 | 2.04 |
| CPED1 | 2.36 |  | GREM1 | 2.22 |  | HIST1H3E | 2.11 |  | HIST2H3D | 2.04 |
| ARHGAP11B; | 2.36 |  | KIAA1211 | 2.20 |  | CDCA5 | 2.11 |  | C9orf116 | 2.04 |
| ARHGAP11A |  |  | CKAP2L | 2.20 |  | ZDHHC19 | 2.11 |  | UNKL | 2.04 |
| CDCA8 | 2.35 |  | INCENP | 2.20 |  | IL13RA2 | 2.11 |  | SPHK1 | 2.04 |
| OCIAD2 | 2.35 |  | C12orf60 | 2.20 |  | C1orf109 | 2.11 |  | SLFN11 | 2.04 |
| GREM1 | 2.35 |  | CENPA | 2.20 |  | TYW5 | 2.11 |  | SIPA1 | 2.04 |
| CLCF1 | 2.35 |  | FAM193B | 2.20 |  | ECT2 | 2.11 |  | CRKL | 2.04 |
| NTN4 | 2.33 |  | CDKN3 | 2.20 |  | GNL3; | 2.10 |  | MPP4 | 2.04 |
| CDS1 | 2.33 |  | CELF2 | 2.19 |  | SNORD19B |  |  | ITPRIPL2 | 2.04 |
| FABP5 | 2.33 |  | TUB | 2.19 |  | SGK3 | 2.10 |  | THAP6 | 2.04 |
| CEL | 2.31 |  | MLLT3 | 2.19 |  | C15orf48; | 2.10 |  | TMEM8A | 2.04 |
| TROAP | 2.31 |  | GALNT6 | 2.19 |  | MIR147B |  |  | MGAT5 | 2.04 |
| ICA1 | 2.31 |  | STX3 | 2.19 |  | SELK | 2.10 |  | STAMBPL1 | 2.03 |
| MICB | 2.31 |  | SLC11A2 | 2.19 |  | DNAJA4 | 2.10 |  | ZNF786 | 2.03 |
| ZC3H12A; | 2.30 |  | B4GALT6 | 2.17 |  | CCDC173 | 2.10 |  | CORO7; | 2.03 |
| MIR6732 |  |  | CSMD2 | 2.17 |  | FBRS | 2.10 |  | PAM16; |  |
| TNFSF4 | 2.30 |  | NRP1 | 2.17 |  | MT1E | 2.10 |  | CORO7-PAM16 |  |
| EMC8 | 2.30 |  | AVIL | 2.17 |  | MRPL55 | 2.08 |  | TYK2 | 2.03 |
| E2F8 | 2.30 |  | NPHP1 | 2.17 |  | ZBTB21 | 2.08 |  | AURKB | 2.03 |
| N4BP2 | 2.30 |  | ATG16L1 | 2.17 |  | DESI1 | 2.08 |  | RAB29 | 2.01 |
| DNMBP | 2.28 |  | MAP2 | 2.17 |  | FAM173A | 2.08 |  | ERICH1 | 2.01 |
| INA | 2.28 |  | GEM | 2.17 |  | KIF2C | 2.08 |  | CKS2 | 2.01 |
| TMC1 | 2.28 |  | NIPAL3 | 2.16 |  | PDGFA | 2.08 |  | C10orf113 | 2.01 |
| HOXC13 | 2.28 |  | LINC00504 | 2.16 |  | GAL | 2.08 |  | WNT5B | 2.01 |
| DOCK10 | 2.28 |  | ZNF823 | 2.16 |  | FXYD5 | 2.08 |  | MLXIP | 2.01 |
| F2RL1 | 2.27 |  | KIAA1524 | 2.14 |  | MYB | 2.08 |  | PNP | 2.01 |
| MFSD6 | 2.27 |  | CPT1A | 2.14 |  | TXLNG | 2.08 |  | WDR1 | 2.01 |
| KIAA1107 | 2.27 |  | SPC25 | 2.14 |  | PRR11 | 2.08 |  | ITPKC | 2.01 |
| ZCCHC2 | 2.27 |  | DNAJC25 | 2.14 |  | C3orf62 | 2.07 |  | VPREB3 | 2.01 |
| NET1 | 2.25 |  | ARHGAP22 | 2.14 |  | TACC3 | 2.07 |  | TSPY8 | 2.01 |
| RTTN | 2.25 |  | ANGPTL6 | 2.14 |  | RFX4 | 2.07 |  | GSG1 | 2.01 |
| OR4F17 | 2.25 |  | IL1A | 2.14 |  | ZNF501 | 2.07 |  | OTOR | 2.01 |

Supplemental Table 1 (continued)

Supplemental Table 1 (continued)

| Factor | Relative Fold Change |  | Factor | Relative Fold Change |  | Factor | Relative Fold Change |  | Factor | Relative Fold Change |
| --- | --- | --- | --- | --- | --- | --- | --- | --- | --- | --- |
| ATG16L1 | 2.01 |  | CCL8 | 1.97 |  | TP53TG3B; | 1.95 |  | ANKRD44 | 1.91 |
| SLC25A37 | 2.01 |  | DIRAS3 | 1.97 |  | TP53TG3C; |  |  | DUSP10 | 1.91 |
| ELK3 | 2.01 |  | ABLIM3 | 1.97 |  | TP53TG3; |  |  | ZCCHC4 | 1.91 |
| OSGEPL1 | 2.01 |  | KANK1 | 1.97 |  | LOC102723655; |  |  | RAB28 | 1.91 |
| TNFAIP3 | 2.01 |  | MT1A | 1.97 |  | hotema; |  |  | DOCK9 | 1.91 |
| PARPBP | 2.01 |  | GLCCI1 | 1.97 |  | RP11-1277H1.5 |  |  | GPR65 | 1.91 |
| ANKRD27 | 2.01 |  | LRRC69; | 1.97 |  | SUMF1 | 1.95 |  | WNK4 | 1.91 |
| SLC19A1 | 2.01 |  | MIR4661 |  |  | GTPBP3 | 1.95 |  | SERPINB2 | 1.91 |
| NUF2 | 2.00 |  | NT5C | 1.97 |  | TRIB1 | 1.95 |  | HYOU1 | 1.91 |
| ZNF385D | 2.00 |  | ETHE1 | 1.96 |  | HPCAL1 | 1.93 |  | SAMD11 | 1.91 |
| ATP10D | 2.00 |  | RGMB | 1.96 |  | MT1G | 1.93 |  | FAM102B | 1.91 |
| NRP1 | 2.00 |  | OAF | 1.96 |  | GPR137B | 1.93 |  | WRAP73 | 1.91 |
| NUSAP1 | 2.00 |  | ZNF324 | 1.96 |  | CCHCR1 | 1.93 |  | USP17L10 | 1.91 |
| IER2 | 2.00 |  | ORC1 | 1.96 |  | PLIN2 | 1.93 |  | MAP3K14 | 1.91 |
| ACPP | 2.00 |  | PKP4 | 1.96 |  | SHANK2-AS3 | 1.93 |  | SMOX | 1.91 |
| LAMP3 | 2.00 |  | NIPAL1 | 1.96 |  | SERPINA9 | 1.93 |  | ATG16L1 | 1.91 |
| FAM196B | 2.00 |  | NAT1 | 1.96 |  | MC4R | 1.93 |  | RPL7A | 1.91 |
| PDK4 | 2.00 |  | TIMELESS | 1.96 |  | SLCO4C1 | 1.93 |  | ATP13A5-AS1 | 1.91 |
| FASN | 2.00 |  | C2orf48 | 1.96 |  | FAM227A | 1.93 |  | FAM117A | 1.91 |
| SYN3 | 2.00 |  | RAB27B | 1.96 |  | SPIB | 1.93 |  | PSG3 | 1.91 |
| OR2T33 | 2.00 |  | GGT2 | 1.96 |  | GALNT14 | 1.93 |  | C17orf58 | 1.91 |
| CYLC2 | 2.00 |  | SEPT1 | 1.96 |  | LYVE1 | 1.93 |  | ZNF792 | 1.91 |
| MVB12A; | 2.00 |  | FAM213B | 1.96 |  | MT1L | 1.93 |  | TMEM178B | 1.91 |
| BISPR |  |  | OR5D14 | 1.96 |  | IKZF3 | 1.93 |  | C19orf12 | 1.91 |
| KIAA1147 | 2.00 |  | ABCD1 | 1.96 |  | ATAD2 | 1.93 |  | RBM45 | 1.89 |
| VEGFC | 1.99 |  | RAI1 | 1.96 |  | SCG5 | 1.93 |  | MYC | 1.89 |
| PDCD1LG2 | 1.99 |  | TBC1D3K | 1.96 |  | SRGAP1 | 1.92 |  | ZBED1;DHRSX | 1.89 |
| MFAP5 | 1.99 |  | FAM129B | 1.96 |  | NEIL3 | 1.92 |  | ZNF506 | 1.89 |
| ANLN | 1.99 |  | IKBKB | 1.95 |  | PSTPIP2 | 1.92 |  | SPDYE5 | 1.89 |
| OLR1 | 1.99 |  | CENPK | 1.95 |  | MROH1 | 1.92 |  | PDE9A | 1.89 |
| H1FNT | 1.99 |  | MRGPRD | 1.95 |  | ZNF774 | 1.92 |  | FCER1A | 1.89 |
| C14orf178 | 1.99 |  | NFATC2IP; | 1.95 |  | DYRK3 | 1.92 |  | KSR2 | 1.89 |
| HNRNPH1 | 1.99 |  | MIR4517 |  |  | TMEM247 | 1.92 |  | FGF9 | 1.89 |
| SETD3 | 1.99 |  | PLEKHG4 | 1.95 |  | ARHGEF3 | 1.92 |  | ACBD6 | 1.89 |
| TCAP | 1.99 |  | ABCA8 | 1.95 |  | HLTF | 1.92 |  | PWP2 | 1.89 |
| ARMC6 | 1.99 |  | CAMKMT | 1.95 |  | NLRP10 | 1.92 |  | EPAS1 | 1.89 |
| ZFP36 | 1.99 |  | TMEM154 | 1.95 |  | OR10K2 | 1.92 |  | PID1 | 1.89 |
| HRH1 | 1.99 |  | PRKAR1B | 1.95 |  | ABCA13 | 1.92 |  | TMEM171 | 1.89 |
| BUD31 | 1.99 |  | ZNF618 | 1.95 |  | WBSCR17 | 1.92 |  | C11orf68 | 1.89 |
| C18orf54 | 1.99 |  | KCNT1 | 1.95 |  | HERC2P3 | 1.92 |  | PAQR5 | 1.89 |
| LIF | 1.99 |  | SFR1 | 1.95 |  | PRKAR1A; | 1.92 |  | UACA | 1.89 |
| MT1X | 1.97 |  | KNTC1 | 1.95 |  | ARSG |  |  | ZNF354B | 1.89 |
| STX1B | 1.97 |  | TP53TG3B | 1.95 |  | SSPN | 1.92 |  | SALL1 | 1.89 |
| DHX33 | 1.97 |  | TP53TG3B; | 1.95 |  | TRIM9 | 1.92 |  | TBC1D3 | 1.89 |
| MTM1 | 1.97 |  | TP53TG3C; |  |  | ATG16L1 | 1.92 |  | ZNF274 | 1.89 |
| MAGEA2; | 1.97 |  | TP53TG3; |  |  | MOGAT1 | 1.92 |  | DPF3 | 1.89 |
| MAGEA2B |  |  | LOC102723655; |  |  | ITGB6 | 1.92 |  | CKAP2 | 1.88 |
| E2F7 | 1.97 |  | RP11-23E10.6 |  |  | C22orf29; | 1.92 |  | UBE2T | 1.88 |
| AURKA | 1.97 |  | NLRC5 | 1.95 |  | GNB1L |  |  | KIAA1211L | 1.88 |
| MANSC1 | 1.97 |  |  |  |  | RAD9A | 1.92 |  | BTBD18 | 1.88 |
| STAB2 | 1.97 |  |  |  |  | ROR1-AS1 | 1.92 |  | C14orf142 | 1.88 |
| PTGES2 | 1.97 |  |  |  |  | XRCC4 | 1.91 |  | AIF1 | 1.88 |
| SPANXB1 | 1.97 |  |  |  |  | ALDH1A3 | 1.91 |  | FAM155B | 1.88 |

| Factor | Relative Fold Change |  | Factor | Relative Fold Change |  | Factor | Relative Fold Change |  | Factor | Relative Fold Change |
| --- | --- | --- | --- | --- | --- | --- | --- | --- | --- | --- |
| NKX3-1 | 1.88 |  | NAV3 | 1.85 |  | SIM2 | 1.83 |  | HSPA1B; | 1.79 |
| CLEC2A | 1.88 |  | USB1 | 1.85 |  | NTNG1 | 1.82 |  | HSPA1A |  |
| SYNE2 | 1.88 |  | RCVRN | 1.85 |  | GALNTL6 | 1.82 |  | CEP85L | 1.79 |
| MST1 | 1.88 |  | LY96 | 1.85 |  | DTX4 | 1.82 |  | ZFPM2 | 1.79 |
| IL31RA | 1.88 |  | SLC20A1 | 1.84 |  | DUS3L | 1.82 |  | KLHL9 | 1.79 |
| JUN | 1.88 |  | ETFDH | 1.84 |  | BIN1 | 1.82 |  | CEP83 | 1.79 |
| ADAMTSL1 | 1.88 |  | SEMA3A | 1.84 |  | F2RL2 | 1.82 |  | CD80 | 1.79 |
| DCAF4 | 1.88 |  | LOC100131315; | 1.84 |  | OR8H3 | 1.82 |  | GPR55 | 1.79 |
| BCR | 1.88 |  | RP11-758N13.1 |  |  | ADRBK1 | 1.82 |  | HIST1H2AL; | 1.79 |
| SOD2 | 1.88 |  | ZNF35 | 1.84 |  | ZBTB38 | 1.82 |  | HIST1H2BN |  |
| MAP3K10 | 1.87 |  | ASB1 | 1.84 |  | LMBR1 | 1.82 |  | HIF3A | 1.79 |
| F10 | 1.87 |  | FAP | 1.84 |  | STC1 | 1.82 |  | WDR4 | 1.79 |
| IRF2 | 1.87 |  | BIRC6 | 1.84 |  | HNRNPF | 1.82 |  | MAN1C1 | 1.79 |
| PEAR1 | 1.87 |  | DDC | 1.84 |  | KCNN4 | 1.82 |  | CDT1 | 1.79 |
| SLC12A7 | 1.87 |  | PTGFR | 1.84 |  | VSTM1 | 1.82 |  | C1orf35 | 1.79 |
| DUSP16 | 1.87 |  | TAAR1 | 1.84 |  | EPN3 | 1.82 |  | HIST1H2AE | 1.79 |
| C1orf105 | 1.87 |  | DSCC1 | 1.84 |  | PNKP | 1.82 |  | ZNF574 | 1.79 |
| PTPRN | 1.87 |  | MORN3 | 1.84 |  | GSDMD | 1.82 |  | PRR12 | 1.79 |
| TNFRSF21 | 1.87 |  | ABCD3 | 1.84 |  | SLC26A4 | 1.82 |  | ARHGAP29 | 1.79 |
| RDH10 | 1.87 |  | CCSAP | 1.84 |  | AKR1B1 | 1.82 |  | RPIA | 1.79 |
| PCDH11X | 1.87 |  | GRB14 | 1.84 |  | TOMM40 | 1.82 |  | E2F3 | 1.79 |
| EIF1AD | 1.87 |  | EDEM1 | 1.84 |  | TADA1 | 1.80 |  | ST7; ST7-OT4; | 1.79 |
| PITPNC1 | 1.87 |  | TPPP | 1.84 |  | MYRF | 1.80 |  | ST7-OT3; |  |
| GLMN | 1.87 |  | SF3B5 | 1.84 |  | FAR2 | 1.80 |  | MIR6132 |  |
| FANCD2 | 1.87 |  | LSAMP | 1.84 |  | TK1 | 1.80 |  | SCARB1 | 1.79 |
| SSH2 | 1.85 |  | IL7R | 1.84 |  | INO80 | 1.80 |  | SLC47A2 | 1.78 |
| PHLDA2 | 1.85 |  | RDH10-AS1 | 1.84 |  | TRIM43 | 1.80 |  | ZNF615 | 1.78 |
| TRAPPC4 | 1.85 |  | EDC4 | 1.84 |  | CRHBP | 1.80 |  | PPARG | 1.78 |
| HHAT | 1.85 |  | TGM2 | 1.84 |  | HECW1 | 1.80 |  | CDX2 | 1.78 |
| OSBPL3 | 1.85 |  | TEX10 | 1.83 |  | VWCE | 1.80 |  | RRP12 | 1.78 |
| TRAPPC4 | 1.85 |  | LACC1 | 1.83 |  | NMS | 1.80 |  | PLD1 | 1.78 |
| ANKRD52 | 1.85 |  | BRINP2 | 1.83 |  | ZKSCAN4 | 1.80 |  | MT2A | 1.78 |
| OVOL2 | 1.85 |  | HIST2H4A; | 1.83 |  | C9orf72 | 1.80 |  | CFC1 | 1.78 |
| SH3BP5L | 1.85 |  | HIST2H4B |  |  | KCNA4 | 1.80 |  | PHF7 | 1.78 |
| TIGD5 | 1.85 |  | STIL | 1.83 |  | CLEC12A | 1.80 |  | PAPD7 | 1.78 |
| IL18 | 1.85 |  | ATP13A3 | 1.83 |  | NEIL1 | 1.80 |  | AIM1 | 1.78 |
| PRIM1 | 1.85 |  | STEAP2 | 1.83 |  | OR10S1 | 1.80 |  | ENPP1 | 1.78 |
| TRIM47 | 1.85 |  | SRGN | 1.83 |  | BLNK | 1.80 |  | NPM2 | 1.78 |
| SULF2 | 1.85 |  | PIK3AP1 | 1.83 |  | FGL2 | 1.80 |  | UXT-AS1 | 1.78 |
| NHSL2 | 1.85 |  | DLAT | 1.83 |  | TCEAL8 | 1.80 |  | ACRC | 1.78 |
| TXNRD1 | 1.85 |  | CBR3 | 1.83 |  | GABRA5 | 1.80 |  | KIAA1551 | 1.78 |
| KIF20B | 1.85 |  | CHIC1 | 1.83 |  | ZNF486 | 1.80 |  | TNFAIP2 | 1.78 |
| TIPARP | 1.85 |  | DUSP5 | 1.83 |  | SLC2A6 | 1.80 |  | SLC22A16 | 1.78 |
| SLC1A3 | 1.85 |  | CCDC85A | 1.83 |  | DNMT3B | 1.80 |  | ADM | 1.78 |
| RPL12 | 1.85 |  | RNFT2 | 1.83 |  | ZNF514 | 1.79 |  | TMEM125 | 1.78 |
| BBX | 1.85 |  | KCNH6 | 1.83 |  | SUFU | 1.79 |  | ADGRF5 | 1.78 |
| GPAT3 | 1.85 |  | WNT7B | 1.83 |  | RFC3 | 1.79 |  | STYXL1 | 1.78 |
| ANK2 | 1.85 |  | GREM2 | 1.83 |  | ADAMTS2 | 1.79 |  | TRH | 1.78 |
| GRB10 | 1.85 |  | FABP2 | 1.83 |  | UPK3BL; | 1.79 |  | RAC3 | 1.78 |
| SLC1A1 | 1.85 |  | PPL | 1.83 |  | POLR2J2 |  |  | TTC7B | 1.77 |
| MELK | 1.85 |  | FCGBP | 1.83 |  | UGCG | 1.79 |  | SEPT10 | 1.77 |
| GNG10 | 1.85 |  | STS | 1.83 |  | TBC1D2B | 1.79 |  | PLD1 | 1.77 |
| ZBED1 | 1.85 |  | RNF128 | 1.83 |  |  |  |  | SIPA1L3 | 1.77 |

Supplemental Table 1 (continued)

| Factor | Relative Fold Change |  | Factor | Relative Fold Change |  | Factor | Relative Fold Change |  | Factor | Relative Fold Change |
| --- | --- | --- | --- | --- | --- | --- | --- | --- | --- | --- |
| CCNB1 | 1.77 |  | F5 | 1.74 |  | MYBL1 | 1.72 |  | ONECUT3 | 1.71 |
| GDF15 | 1.77 |  | CACNA1A | 1.74 |  | ARHGAP26 | 1.72 |  | ZNF749 | 1.71 |
| C8orf33 | 1.77 |  | TRIP13 | 1.74 |  | DUSP26 | 1.72 |  | GSG2 | 1.71 |
| GAS2L3 | 1.77 |  | SLC2A8 | 1.74 |  | STK32C | 1.72 |  | C5orf30 | 1.71 |
| OR5A2 | 1.77 |  | CDK5RAP2 | 1.74 |  | LECT1 | 1.72 |  | ARHGEF10 | 1.71 |
| GJD2 | 1.77 |  | FOXP3 | 1.74 |  | OR4A8 | 1.72 |  | LOC646938 | 1.71 |
| MC5R | 1.77 |  | RAD51B | 1.74 |  | OR52N1 | 1.72 |  | ABHD3 | 1.71 |
| S1PR1 | 1.77 |  | TAOK2 | 1.74 |  | TAS2R30; | 1.72 |  | KIAA1033 | 1.71 |
| POC1A | 1.77 |  | KLHDC4 | 1.74 |  | TAS2R43; |  |  | ZNF358 | 1.71 |
| CALB2 | 1.77 |  | CYP2F1 | 1.74 |  | PRR4 |  |  | HIST2H2AB | 1.71 |
| SPG7 | 1.77 |  | MAFF | 1.74 |  | CD8B | 1.72 |  | CHORDC1 | 1.71 |
| CERS3 | 1.77 |  | AADACL3 | 1.74 |  | NMUR2 | 1.72 |  | ZNF71 | 1.69 |
| PRSS56 | 1.77 |  | CHRM3 | 1.74 |  | RBMXL3 | 1.72 |  | PPP6R1 | 1.69 |
| MOK | 1.77 |  | MAP2K3 | 1.74 |  | ZNF646 | 1.72 |  | SERPINE2 | 1.69 |
| CDC42EP4 | 1.77 |  | TMEM161A | 1.74 |  | FAM72B | 1.72 |  | PHLDA1 | 1.69 |
| CENPH | 1.77 |  | LRRC20 | 1.74 |  | ATG4B | 1.72 |  | KCNG1 | 1.69 |
| DEFB106A | 1.77 |  | SPDYE1 | 1.74 |  | PTPRE | 1.72 |  | CYP20A1 | 1.69 |
| AQP3 | 1.77 |  | FZD9 | 1.74 |  | P3H3 | 1.72 |  | MGST1 | 1.69 |
| OR52E4 | 1.77 |  | MRPL44 | 1.74 |  | CPNE2 | 1.72 |  | GPR3 | 1.69 |
| ZBTB3 | 1.77 |  | GOLGA4 | 1.73 |  | MKKS | 1.72 |  | APBA1 | 1.69 |
| FOSB | 1.77 |  | WDHD1 | 1.73 |  | LIMS3L | 1.72 |  | KRTAP5-AS1 | 1.69 |
| LGALS4 | 1.77 |  | PARP12 | 1.73 |  | ARFGEF3 | 1.72 |  | ACAP2; | 1.69 |
| ZNF835 | 1.77 |  | FZD2 | 1.73 |  | CARHSP1 | 1.72 |  | ACAP2-IT1; |  |
| COL10A1 | 1.77 |  | SLC25A22 | 1.73 |  | TGIF1 | 1.71 |  | AC090018.3 |  |
| SLC6A15 | 1.77 |  | HIST1H4L | 1.73 |  | HACD4 | 1.71 |  | SYK | 1.69 |
| PDS5B | 1.77 |  | KIF22 | 1.73 |  | MMP16 | 1.71 |  | SPRED2 | 1.69 |
| MBP | 1.77 |  | VPS9D1 | 1.73 |  | RGS3 | 1.71 |  | C7orf34 | 1.69 |
| HGS | 1.77 |  | EVI2A; EVI2B | 1.73 |  | TRIM38 | 1.71 |  | KLF4 | 1.69 |
| GBP3 | 1.77 |  | APOB | 1.73 |  | RFESD | 1.71 |  | ANKRD30A | 1.69 |
| FLNA | 1.75 |  | PDE10A | 1.73 |  | JAK2 | 1.71 |  | FOXI2 | 1.69 |
| ACADSB | 1.75 |  | TP53TG3C | 1.73 |  | RRBP1 | 1.71 |  | CRTAC1 | 1.69 |
| CYP24A1 | 1.75 |  | ALG1L2 | 1.73 |  | ZNF669 | 1.71 |  | PATE3 | 1.69 |
| USP1 | 1.75 |  | ROPN1L | 1.73 |  | TLR1 | 1.71 |  | CIB1 | 1.69 |
| ARHGEF11 | 1.75 |  | NCAM1 | 1.73 |  | GLRX | 1.71 |  | MLXIP | 1.69 |
| NOC4L | 1.75 |  | SLC38A8 | 1.73 |  | FGF1 | 1.71 |  | ZNF197 | 1.69 |
| ADH4 | 1.75 |  | ZNF780A | 1.73 |  | DAXX | 1.71 |  | ATP1A1 | 1.69 |
| OR5D16 | 1.75 |  | GPATCH2 | 1.73 |  | SNX10 | 1.71 |  | SPDYA | 1.69 |
| AMIGO3 | 1.75 |  | OR5K4 | 1.73 |  | WDR86 | 1.71 |  | NOP14 | 1.69 |
| KCND2 | 1.75 |  | PNMA3 | 1.73 |  | MID1IP1 | 1.71 |  | MUC22 | 1.69 |
| TP53INP2 | 1.75 |  | KIF11 | 1.73 |  | TAF1 | 1.71 |  | AGO2 | 1.69 |
| POTEI | 1.75 |  | TRIM49 | 1.73 |  | ZNF720 | 1.71 |  | ENO4 | 1.69 |
| CFAP46 | 1.75 |  | KIF21A | 1.73 |  | TOX2 | 1.71 |  | BEAN1 | 1.69 |
| GYLTL1B | 1.75 |  | ZNF652 | 1.73 |  | RPAP2 | 1.71 |  | NPIPB6 | 1.69 |
| RASAL3 | 1.75 |  | MUC7 | 1.73 |  | NCF2 | 1.71 |  | HOXB2 | 1.69 |
| TM4SF19 | 1.75 |  | ZBED8 | 1.73 |  | ZBTB11 | 1.71 |  | RPS6KA1 | 1.69 |
| OR2L13 | 1.75 |  | MSL2 | 1.73 |  | PPP1R11 | 1.71 |  | TMEM39B | 1.69 |
| BRD1 | 1.75 |  | NOL6 | 1.73 |  | MMP13 | 1.71 |  | TMIE | 1.69 |
| MFSD2A | 1.75 |  | FAM134A | 1.72 |  | SMURF2 | 1.71 |  | PKHD1L1 | 1.69 |
| MEA1 | 1.75 |  | VEZT | 1.72 |  | PMAIP1 | 1.71 |  | ZNF345 | 1.69 |
| PRPF19 | 1.75 |  | MT1B; MT1CP | 1.72 |  | VPS8 | 1.71 |  | ZNF672 | 1.69 |
| NCR3LG1 | 1.74 |  | SPTLC2 | 1.72 |  | CCDC191 | 1.71 |  | CD9 | 1.69 |
| POR; MIR4651 | 1.74 |  | CCDC13 | 1.72 |  | WIPF3 | 1.71 |  | TRAIP | 1.68 |
| POGZ | 1.74 |  | TMEM206 | 1.72 |  | WDR81 | 1.71 |  | TEX101 | 1.68 |

Supplemental Table 1 (continued)

| Factor | Relative Fold Change |  | Factor | Relative Fold Change |  | Factor | Relative Fold Change |  | Factor | Relative Fold Change |
| --- | --- | --- | --- | --- | --- | --- | --- | --- | --- | --- |
| AMPD2 | 1.68 |  | ZGPAT; LIME1 | 1.67 |  | POLR3K | 1.66 |  | MRPL48 | 1.64 |
| DOK7 | 1.68 |  | MIEF1 | 1.67 |  | TRPV2 | 1.66 |  | TMBIM6 | 1.64 |
| CLK4 | 1.68 |  | GNAT2 | 1.67 |  | ZNF714; | 1.66 |  | CD96 | 1.64 |
| KITLG | 1.68 |  | DNAH5 | 1.67 |  | VN1R81P |  |  | LAMC3 | 1.64 |
| FLOT2 | 1.68 |  | TAAR8 | 1.67 |  | LAMA3 | 1.66 |  | VIM | 1.64 |
| MGAT4C | 1.68 |  | DNAJC9 | 1.67 |  | TNXA | 1.66 |  | LOC100652768; | 1.64 |
| CCL16 | 1.68 |  | PCDH9 | 1.67 |  | CPAMD8 | 1.66 |  | PCSK7 |  |
| NEK2 | 1.68 |  | ABCC4 | 1.67 |  | TMEM158 | 1.66 |  | HAS3 | 1.64 |
| KLHL29 | 1.68 |  | OR2C1 | 1.67 |  | GOLGA3 | 1.66 |  | NOL4 | 1.64 |
| CNRIP1 | 1.68 |  | PARP15 | 1.67 |  | PPIE | 1.66 |  | EFNA2 | 1.64 |
| GJD4 | 1.68 |  | LRRD1 | 1.67 |  | BCL2L11 | 1.66 |  | NDUFV3 | 1.64 |
| CFAP43 | 1.68 |  | ADCY8 | 1.67 |  | KCNJ8 | 1.66 |  | S100A5 | 1.64 |
| OR4C12 | 1.68 |  | C12orf42 | 1.67 |  | PNPLA3 | 1.66 |  | BRPF1 | 1.64 |
| PRKCH | 1.68 |  | CENPJ | 1.67 |  | PPIF | 1.65 |  | ATR | 1.64 |
| ZNF446 | 1.68 |  | LOC388282; | 1.67 |  | SPRYD4 | 1.65 |  | TRPC7 | 1.64 |
| C2orf80 | 1.68 |  | CTD-2600O9.1 |  |  | TMEM170A | 1.65 |  | TAT | 1.64 |
| EXOSC6 | 1.68 |  | MAB21L3 | 1.67 |  | CRELD2 | 1.65 |  | IRAK1; MIR718 | 1.64 |
| CRAMP1 | 1.68 |  | YARS | 1.67 |  | IL1RL2 | 1.65 |  | CC2D1A | 1.64 |
| MED25; | 1.68 |  | GRM7 | 1.67 |  | HEATR5A | 1.65 |  | GDNF | 1.64 |
| MIR6800 |  |  | SHROOM2 | 1.67 |  | HIPK1 | 1.65 |  | GFPT2 | 1.64 |
| AHDC1 | 1.68 |  | DOC2A | 1.67 |  | MERTK | 1.65 |  | FBXL14 | 1.64 |
| C2orf44 | 1.68 |  | DEFB116 | 1.67 |  | ARSH | 1.65 |  | PTHLH | 1.64 |
| SH3RF1 | 1.68 |  | ZNF502 | 1.67 |  | GLYATL1 | 1.65 |  | RAB8B | 1.64 |
| MYO10 | 1.68 |  | AQP11 | 1.67 |  | ADPRH | 1.65 |  | AKAP8 | 1.64 |
| DMGDH | 1.68 |  | NARFL | 1.67 |  | MUC13 | 1.65 |  | EPT1 | 1.64 |
| FADS2 | 1.68 |  | SOCS6 | 1.67 |  | FAM26F | 1.65 |  | FABP5 | 1.64 |
| QPCTL | 1.68 |  | UBE2S | 1.67 |  | HLA-DOA | 1.65 |  | AMIGO2 | 1.62 |
| NDUFB7 | 1.68 |  | SP100 | 1.67 |  | CSPG4P1Y; | 1.65 |  | ZBTB7A | 1.62 |
| ACOT8 | 1.68 |  | TNPO2 | 1.67 |  | DNM1P24 |  |  | LSM11 | 1.62 |
| TRMT13 | 1.67 |  | PRR16 | 1.66 |  | ANK3 | 1.65 |  | ANO2 | 1.62 |
| TMEM53 | 1.67 |  | CCNJ | 1.66 |  | CHST11 | 1.65 |  | CHTOP | 1.62 |
| SS18L2 | 1.67 |  | NR1D1 | 1.66 |  | SOX21 | 1.65 |  | F13B | 1.62 |
| IRF1 | 1.67 |  | LRRC49 | 1.66 |  | ADAMTS5 | 1.65 |  | VPS54 | 1.62 |
| FGD2 | 1.67 |  | HNRNPCL4 | 1.66 |  | WNT5A | 1.65 |  | ABI3BP | 1.62 |
| HNF4G | 1.67 |  | ETV4 | 1.66 |  | KRBOX1 | 1.65 |  | DNAAF5 | 1.62 |
| STRBP; | 1.67 |  | C9orf152 | 1.66 |  | MAL2 | 1.65 |  | LOC389602; | 1.62 |
| MIR600HG; |  |  | ZNF229 | 1.66 |  | CCDC169- | 1.65 |  | AC021218.2 |  |
| MIR600 |  |  | MIR670HG; | 1.66 |  | SOHLH2 |  |  | TIAL1 | 1.62 |
| FADS1; | 1.67 |  | HSD17B12 |  |  | STXBP6 | 1.65 |  | OR6Q1 | 1.62 |
| MIR1908 |  |  | IRX5 | 1.66 |  | NFKBID | 1.65 |  | OR4M2; | 1.62 |
| ZPR1 | 1.67 |  | OR2K2 | 1.66 |  | ZDHHC3 | 1.65 |  | AC171558.2 |  |
| C12orf45 | 1.67 |  | BDNF | 1.66 |  | GID4 | 1.65 |  | SLC25A1 | 1.62 |
| VDR | 1.67 |  | FAM216A | 1.66 |  | RBCK1 | 1.65 |  | CACNA1D | 1.62 |
| RACGAP1 | 1.67 |  | ZNF8 | 1.66 |  | OSMR | 1.64 |  | C3orf18 | 1.62 |
| APH1B | 1.67 |  | ART3 | 1.66 |  | ATP13A3 | 1.64 |  | UVSSA | 1.62 |
| VIMP | 1.67 |  | RXFP3 | 1.66 |  | TBC1D9 | 1.64 |  | UTP3 | 1.62 |
| ITPA | 1.67 |  | PCDHB6 | 1.66 |  | TMEM192 | 1.64 |  | GABRA6 | 1.62 |
| DNAJC5; | 1.67 |  | PAEP | 1.66 |  | RPP21 | 1.64 |  | TNXB | 1.62 |
| MIR941-1; |  |  | IPPK | 1.66 |  | SH2B3 | 1.64 |  | TAP2 | 1.62 |
| MIR941-2; |  |  | OR2D2 | 1.66 |  | RAD52 | 1.64 |  | ZBTB43 | 1.62 |
| MIR941-3; |  |  | CCDC34 | 1.66 |  | PROCR | 1.64 |  | LAS1L | 1.62 |
| MIR941-4; |  |  | KRT6C | 1.66 |  | HOXA7 | 1.64 |  | TMEM26 | 1.62 |
| MIR941-5 |  |  | TBX3 | 1.66 |  | RPS6KA4 | 1.64 |  | ACBD7 | 1.62 |

Supplemental Table 1 (continued)

| Factor | Relative Fold Change |  | Factor | Relative Fold Change |  | Factor | Relative Fold Change |  | Factor | Relative Fold Change |
| --- | --- | --- | --- | --- | --- | --- | --- | --- | --- | --- |
| TYR | 1.62 |  | FAM210A | 1.61 |  | RHBDL3 | 1.60 |  | IQCF3 | 1.58 |
| ZC3H12C | 1.62 |  | ABHD3 | 1.61 |  | GRK6 | 1.60 |  | ZNF239 | 1.58 |
| ANO6 | 1.62 |  | ADAMTS10 | 1.61 |  | PPP1R18 | 1.60 |  | OR5D13 | 1.58 |
| DNAJC14 | 1.62 |  | RPTOR | 1.61 |  | ZNF705B | 1.60 |  | FOXN4 | 1.58 |
| RILP | 1.62 |  | GIPC1 | 1.61 |  | SLC10A5 | 1.60 |  | ZNF891 | 1.58 |
| SYNDIG1 | 1.62 |  | HTR1D | 1.61 |  | DSP | 1.59 |  | NMRAL1 | 1.58 |
| LINC01270 | 1.62 |  | ATP13A2 | 1.61 |  | RAD23B | 1.59 |  | REM1 | 1.58 |
| PATZ1 | 1.62 |  | NAALADL1 | 1.61 |  | TUBGCP3 | 1.59 |  | TMCO4 | 1.58 |
| BCL2L14 | 1.62 |  | DNM1L | 1.61 |  | ZNF730 | 1.59 |  | EAF2 | 1.58 |
| LY6G6D | 1.62 |  | RAC2 | 1.61 |  | PDIK1L | 1.59 |  | TRAPPC4 | 1.58 |
| ABCC9 | 1.62 |  | PDIA4 | 1.61 |  | DENND2D | 1.59 |  | L3MBTL1 | 1.58 |
| ALDH1A2 | 1.62 |  | SKI | 1.60 |  | NMNAT2 | 1.59 |  | WNT10A | 1.58 |
| OR5F1 | 1.62 |  | HELQ | 1.60 |  | C3orf67 | 1.59 |  | KLHL41 | 1.58 |
| UNC119B | 1.62 |  | THSD7A | 1.60 |  | MECOM | 1.59 |  | AGGF1 | 1.58 |
| LPAR5 | 1.62 |  | MAGEE1 | 1.60 |  | NCEH1 | 1.59 |  | GRM6 | 1.58 |
| FKBP11; ARF3 | 1.62 |  | PRRT2 | 1.60 |  | C6orf223 | 1.59 |  | ZMIZ2 | 1.58 |
| PGS1 | 1.62 |  | TBC1D3C | 1.60 |  | HGSNAT | 1.59 |  | RAMP3 | 1.58 |
| SMYD3 | 1.62 |  | TYMS | 1.60 |  | SLC25A51 | 1.59 |  | CCDC136 | 1.58 |
| RPP14 | 1.62 |  | RASA3 | 1.60 |  | CLIC2 | 1.59 |  | CELF2 | 1.58 |
| UHRF2 | 1.62 |  | C6orf1 | 1.60 |  | ADAMTS14 | 1.59 |  | LRRC3C | 1.58 |
| SSNA1 | 1.62 |  | HSFX1 | 1.60 |  | LIPA | 1.59 |  | TMIGD1 | 1.58 |
| DRAXIN | 1.61 |  | LRRC23 | 1.60 |  | MPHOSPH6 | 1.59 |  | DOHH | 1.58 |
| NCKAP5 | 1.61 |  | SAG | 1.60 |  | RARA | 1.59 |  | CD40 | 1.58 |
| PLEKHG4B | 1.61 |  | GMPPB | 1.60 |  | HAP1 | 1.59 |  | DMC1 | 1.58 |
| SALL2 | 1.61 |  | DAZ4; DAZ1 | 1.60 |  | SGSH | 1.59 |  | OR2T5 | 1.58 |
| CKS1B | 1.61 |  | DAZ3 | 1.60 |  | OAZ1 | 1.59 |  | TRIM17 | 1.58 |
| XPO5 | 1.61 |  | HHEX | 1.60 |  | EPS15L1 | 1.59 |  | BUB1 | 1.58 |
| NINL | 1.61 |  | C14orf159 | 1.60 |  | CHRDL1 | 1.59 |  | TIPARP | 1.58 |
| ADPRHL2 | 1.61 |  | TMCO5A | 1.60 |  | GSTO1 | 1.59 |  | EIF4G1 | 1.58 |
| CENPL | 1.61 |  | C15orf54 | 1.60 |  | FBXW4 | 1.59 |  | SRPRB | 1.58 |
| EBLN2 | 1.61 |  | POTEB | 1.60 |  | POLE2 | 1.59 |  | ACSL6 | 1.58 |
| HABP4 | 1.61 |  | ONECUT1 | 1.60 |  | CA12 | 1.59 |  | BMPER | 1.58 |
| PGR | 1.61 |  | MUC20; | 1.60 |  | C16orf52 | 1.59 |  | RP11-875O11.1; | 1.58 |
| CCDC153 | 1.61 |  | SDHAP2; |  |  | TULP2 | 1.59 |  | RHOBTB2 |  |
| ARHGAP17 | 1.61 |  | MIR570; |  |  | CXADR | 1.59 |  | MTFR1 | 1.58 |
| SIGLEC11 | 1.61 |  | LINC00969 |  |  | RHEBL1 | 1.59 |  | MSANTD3 | 1.58 |
| LEKR1 | 1.61 |  | LOC650293 | 1.60 |  | IL1RL1 | 1.59 |  | PUDP | 1.58 |
| RGS6 | 1.61 |  | ZFPM2 | 1.60 |  | MGLL | 1.59 |  | B4GALNT4 | 1.58 |
| CDC25C | 1.61 |  | FOXO4 | 1.60 |  | KIFC1 | 1.59 |  | ZW10 | 1.58 |
| HSPH1 | 1.61 |  | SFMBT2 | 1.60 |  | MB21D1 | 1.59 |  | ZNF26 | 1.58 |
| ZFP30 | 1.61 |  | SLC16A12 | 1.60 |  | LOC101927550; | 1.59 |  | CYSLTR2 | 1.58 |
| DENND4B | 1.61 |  | SORL1 | 1.60 |  | AC004893.11; |  |  | FLYWCH1 | 1.58 |
| ACVR1C | 1.61 |  | ZNF197 | 1.60 |  | SMURF1 |  |  | INPP5K | 1.58 |
| SPATA16 | 1.61 |  | CYP3A7; | 1.60 |  | OR4Q2 | 1.59 |  | C18orf63 | 1.58 |
| CLDN1 | 1.61 |  | CYP3A7- |  |  | OCLM | 1.59 |  | ANGPTL4 | 1.58 |
| KLF10 | 1.61 |  | CYP3A51P |  |  | SH2B3 | 1.59 |  | ZSCAN22; | 1.58 |
| C5 | 1.61 |  | SDR42E2 | 1.60 |  | TFPI | 1.59 |  | MIR6806 |  |
| TIMM8A | 1.61 |  | TMA16 | 1.60 |  | NCAPG2 | 1.59 |  | STK35 | 1.58 |
| USP9Y;TTTY15 | 1.61 |  | LPCAT1 | 1.60 |  | BCL2L1 | 1.59 |  | ZKSCAN7 | 1.58 |
| TTTY15 |  |  | DIAPH1 | 1.60 |  | SH3BP5 | 1.58 |  | GYPA | 1.58 |
| SPATS2 | 1.61 |  | KIF23 | 1.60 |  | LOC100133091 | 1.58 |  | AMPD3 | 1.58 |
| GANC | 1.61 |  | NPIPB8 | 1.60 |  | JMJD1C | 1.58 |  | AK7 | 1.58 |
| IRX3 | 1.61 |  | NETO2 | 1.60 |  | HTATIP2 | 1.58 |  | SH2B1 | 1.58 |

Supplemental Table 1 (continued)

| Factor | Relative Fold Change |  | Factor | Relative Fold Change |  | Factor | Relative Fold Change |  | Factor | Relative Fold Change |
| --- | --- | --- | --- | --- | --- | --- | --- | --- | --- | --- |
| NETO1 | 1.58 |  | GPSM1 | 1.56 |  | LILRB5 | 1.55 |  | ATP2B1 | 1.55 |
| KRTAP2-3 | 1.58 |  | UQCC3 | 1.56 |  | RHOH | 1.55 |  | DYNC1H1 | 1.55 |
| KMT2D | 1.58 |  | NUP58 | 1.56 |  | KLHL3 | 1.55 |  | SLC36A4 | 1.54 |
| PHF19 | 1.58 |  | ISLR2 | 1.56 |  | PSMB8-AS1 | 1.55 |  | SCARA3 | 1.54 |
| LAMC2 | 1.57 |  | RAB11FIP3 | 1.56 |  | MGAM | 1.55 |  | RSC1A1; DDI2 | 1.54 |
| RALGPS2 | 1.57 |  | FOXN2 | 1.56 |  | CLUH | 1.55 |  | CARD19 | 1.54 |
| ELMOD3 | 1.57 |  | MEPE | 1.56 |  | DNM2 | 1.55 |  | TUBB4B | 1.54 |
| ADCYAP1 | 1.57 |  | FAM50A | 1.56 |  | ECM1 | 1.55 |  | PGM2L1 | 1.54 |
| TTL | 1.57 |  | CCDC172 | 1.56 |  | AKR7A3 | 1.55 |  | WDR83 | 1.54 |
| KIAA0368 | 1.57 |  | TMCC3; | 1.56 |  | RSRP1 | 1.55 |  | CHST3 | 1.54 |
| TTC36 | 1.57 |  | MIR7844 |  |  | LPIN1 | 1.55 |  | OR2T27 | 1.54 |
| RNF111 | 1.57 |  | DENND4A | 1.56 |  | PARD3B | 1.55 |  | OR52I1 | 1.54 |
| C19orf70 | 1.57 |  | PSKH1 | 1.56 |  | MCM6 | 1.55 |  | GATAD2A | 1.54 |
| FEN1 | 1.57 |  | HMBS | 1.56 |  | GATA2 | 1.55 |  | GMNC | 1.54 |
| FAM151B | 1.57 |  | SKI; kerlabu | 1.56 |  | CDKN2AIP | 1.55 |  | NELL1 | 1.54 |
| RFESD | 1.57 |  | FLOT1 | 1.56 |  | MAD2L1 | 1.55 |  | CLEC1B | 1.54 |
| TRMT2A | 1.57 |  | RADIL | 1.56 |  | SLC30A5 | 1.55 |  | DIO3 | 1.54 |
| HIST1H2BE | 1.57 |  | PEBP4 | 1.56 |  | GNMT | 1.55 |  | HNF1B | 1.54 |
| CLDN2 | 1.57 |  | THSD4 | 1.56 |  | KATNA1 | 1.55 |  | HMBS | 1.54 |
| TSSC4 | 1.57 |  | KIRREL2 | 1.56 |  | DNAJB9 | 1.55 |  | CALML6 | 1.54 |
| SFTA3 | 1.57 |  | SEC16B | 1.56 |  | LACTB2 | 1.55 |  | ZSCAN20 | 1.54 |
| ZNF785 | 1.57 |  | CCDC96 | 1.56 |  | TMOD1 | 1.55 |  | CERKL | 1.54 |
| USP29 | 1.57 |  | MCF2 | 1.56 |  | ALDOB | 1.55 |  | RUFY1 | 1.54 |
| PSD4 | 1.57 |  | ORAOV1 | 1.56 |  | SMIM10L2A | 1.55 |  | CD83 | 1.54 |
| OR8H2 | 1.57 |  | CEP76 | 1.56 |  | TMEM72 | 1.55 |  | ORC6 | 1.54 |
| IZUMO1 | 1.57 |  | DLX1 | 1.56 |  | B3GAT1 | 1.55 |  | JMJD6 | 1.54 |
| CASQ2 | 1.57 |  | MXD1 | 1.56 |  | RFC5 | 1.55 |  | SWSAP1 | 1.54 |
| OR8B2 | 1.57 |  | NEUROD6 | 1.56 |  | FGD6 | 1.55 |  | CTPS1 | 1.54 |
| ARHGEF2 | 1.57 |  | USP51 | 1.56 |  | KLRC2 | 1.55 |  | TLL1 | 1.54 |
| ATP8A1 | 1.57 |  | RRAD | 1.56 |  | POU4F1 | 1.55 |  | SPN | 1.54 |
| KCNQ5 | 1.57 |  | GNA15 | 1.56 |  | THSD4 | 1.55 |  | TBC1D24 | 1.54 |
| TAS2R60 | 1.57 |  | THAP8 | 1.56 |  | NEDD4 | 1.55 |  | MAP4K1 | 1.54 |
| FXN | 1.57 |  | EPO | 1.56 |  | PDCD7 | 1.55 |  | BTG2 | 1.54 |
| SH2D1A | 1.57 |  | CCNE2 | 1.56 |  | TCEB3CL; | 1.55 |  | LRP8 | 1.54 |
| AKAP17A; | 1.57 |  | PLCB1 | 1.56 |  | TCEB3C |  |  | LINC01140 | 1.54 |
| SFRS17A |  |  | MALL | 1.56 |  | ZNF681 | 1.55 |  | SNRPC | 1.54 |
| PTPRR | 1.57 |  | ABHD6 | 1.56 |  | DPH3P1 | 1.55 |  | ABRACL | 1.54 |
| EDNRB | 1.57 |  | TMEM234 | 1.56 |  | YDJC | 1.55 |  | SSC4D | 1.54 |
| EML1 | 1.57 |  | OR2J2 | 1.56 |  | ATIC | 1.55 |  | PPRC1 | 1.54 |
| TMEM97 | 1.57 |  | PNPLA8 | 1.56 |  | MXD1 | 1.55 |  | MSANTD2 | 1.54 |
| TRAPPC4 | 1.57 |  | TRMT10B | 1.56 |  | OR51A2 | 1.55 |  | MEDAG | 1.54 |
| KCNQ5 | 1.57 |  | TXN | 1.56 |  | HBE1 | 1.55 |  | BNC1 | 1.54 |
| NSMAF | 1.57 |  | CALML3 | 1.56 |  | FOXC2 | 1.55 |  | VPS4A | 1.54 |
| MRPL13 | 1.57 |  | GTF2H3 | 1.56 |  | LOC100130880; | 1.55 |  | WDR18 | 1.54 |
| MRPS30 | 1.57 |  | CASP1 | 1.56 |  | AC022173.2 |  |  | ZNF526 | 1.54 |
| KIAA0513 | 1.57 |  | RBMX; | 1.55 |  | VGLL1 | 1.55 |  | HOMER3 | 1.54 |
| HES4 | 1.56 |  | SNORD61 |  |  | PSMC3IP | 1.55 |  | UBE3A | 1.54 |
| THG1L | 1.56 |  | C5orf42 | 1.55 |  | OMG | 1.55 |  | GINS3 | 1.54 |
| ECHDC1 | 1.56 |  | NUP153 | 1.55 |  | NSUN6 | 1.55 |  | SLC2A6 | 1.54 |
| ERO1A | 1.56 |  | BZW2 | 1.55 |  | OLFML2B | 1.55 |  | BTBD17 | 1.54 |
| KCNA2 | 1.56 |  | H2BFM | 1.55 |  | SEC24A | 1.55 |  | DPP10 | 1.54 |
| DUSP7 | 1.56 |  | KDM5A | 1.55 |  | LSM8 | 1.55 |  | SH3BGRL2 | 1.54 |
| SRRM3 | 1.56 |  | ZNF517 | 1.55 |  | NAA10 | 1.55 |  | POLR2J3 | 1.54 |

Supplemental Table 1 (continued)

| Factor | Relative Fold Change |  | Factor | Relative Fold Change |  | Factor | Relative Fold Change |  | Factor | Relative Fold Change |
| --- | --- | --- | --- | --- | --- | --- | --- | --- | --- | --- |
| LOC101060389; | 1.54 |  | TBC1D16 | 1.53 |  | ZC3H8 | 1.52 |  | HERC3 | 1.51 |
| TBC1D3D |  |  | ANKRD37 | 1.53 |  | CCM2 | 1.52 |  | FABP4 | 1.51 |
| LYSMD3 | 1.53 |  | APBB2 | 1.53 |  | ZC3HAV1 | 1.52 |  | CLN8 | 1.51 |
| FICD | 1.53 |  | SIMC1 | 1.53 |  | LINC00311 | 1.52 |  | APBB1IP | 1.51 |
| LSM4 | 1.53 |  | CCDC59 | 1.53 |  | NTAN1 | 1.52 |  | MUC2 | 1.51 |
| SMC6 | 1.53 |  | FGD4 | 1.53 |  | FOPNL | 1.52 |  | MAP3K3 | 1.51 |
| MTMR14 | 1.53 |  | GAS8 | 1.53 |  | RHBDF2 | 1.52 |  | ZNF573 | 1.51 |
| TM2D2 | 1.53 |  | RP11-93O14.2; | 1.53 |  | CRTAM | 1.52 |  | RCC1; | 1.51 |
| CAT | 1.53 |  | VPS35 |  |  | MCTP2 | 1.52 |  | SNORA73A; |  |
| CD3EAP | 1.53 |  | TBCD | 1.53 |  | ZNF213 | 1.52 |  | SNHG3; |  |
| PLEKHA2 | 1.53 |  | C18orf21 | 1.53 |  | BCL2L15 | 1.52 |  | SNORA73B |  |
| KIN | 1.53 |  | REXO1; | 1.53 |  | BTC | 1.52 |  | TAF12 | 1.51 |
| KAT5 | 1.53 |  | MIR1909 |  |  | ZNF707 | 1.52 |  | GCLM | 1.51 |
| ASCL2 | 1.53 |  | TRAPPC4 | 1.53 |  | XKRY2; XKRY | 1.52 |  | NUAK2 | 1.51 |
| NUP37 | 1.53 |  | LIN9 | 1.53 |  | MINOS1-NBL1 | 1.52 |  | PQLC2L | 1.51 |
| DCT | 1.53 |  | ESCO1 | 1.53 |  | SLC2A6 | 1.52 |  | ST3GAL6 | 1.51 |
| KRTAP9-4 | 1.53 |  | PDPR | 1.52 |  | SKOR1 | 1.52 |  | USP38 | 1.51 |
| TIPRL | 1.53 |  | FAM72D | 1.52 |  | UBN1 | 1.52 |  | ADRB2 | 1.51 |
| BDH1 | 1.53 |  | CHRNA10; | 1.52 |  | SLC2A6 | 1.52 |  | CDH10 | 1.51 |
| EMB | 1.53 |  | NUP98 |  |  | SNIP1 | 1.52 |  | NUP155 | 1.51 |
| EBF1 | 1.53 |  | ZNF721 | 1.52 |  | MYO5A | 1.52 |  | MBLAC2 | 1.51 |
| STXBP5 | 1.53 |  | ZNF638 | 1.52 |  | LINS1 | 1.52 |  | HIST1H3I | 1.51 |
| RENBP | 1.53 |  | ATG16L1 | 1.52 |  | NPC1 | 1.52 |  | FGFR1OP | 1.51 |
| OR5M10 | 1.53 |  | AKR1C2 | 1.52 |  | KNCN | 1.51 |  | AKR1B10 | 1.51 |
| CREBZF | 1.53 |  | GPR160 | 1.52 |  | USP37 | 1.51 |  | ZFAND2A | 1.51 |
| FAM174B | 1.53 |  | ALS2CL | 1.52 |  | OPCML | 1.51 |  | CLDN12 | 1.51 |
| SPG7 | 1.53 |  | MAK | 1.52 |  | OTUB1 | 1.51 |  | TSPAN7 | 1.51 |
| KIF16B | 1.53 |  | DRP2 | 1.52 |  | IMP4 | 1.51 |  | OGDHL | 1.51 |
| NOS1 | 1.53 |  | NPR1 | 1.52 |  | COL27A1 | 1.51 |  | CDHR5 | 1.51 |
| SYT4 | 1.53 |  | TIMM17A | 1.52 |  | CTSC | 1.51 |  | HCAR1 | 1.51 |
| CGREF1 | 1.53 |  | TACSTD2 | 1.52 |  | POLR3G | 1.51 |  | ENOX1 | 1.51 |
| MXD4 | 1.53 |  | CAD | 1.52 |  | GOLGA6B | 1.51 |  | TMEM219 | 1.51 |
| FAM46D | 1.53 |  | LIPT1 | 1.52 |  | OXCT2P1 | 1.51 |  | NOTUM | 1.51 |
| IAPP | 1.53 |  | HIST1H1T | 1.52 |  | ABCD3 | 1.51 |  | ABR | 1.51 |
| NAPSA | 1.53 |  | PLAG1 | 1.52 |  | BTN2A3P | 1.51 |  | FFAR3 | 1.51 |
| FAM84A | 1.53 |  | SDR16C5 | 1.52 |  | RGS17 | 1.51 |  | MED29 | 1.51 |
| SPEG | 1.53 |  | PRRG3 | 1.52 |  | SLURP1 | 1.51 |  | CRYBB1 | 1.51 |
| WDR86-AS1 | 1.53 |  | CHCHD1 | 1.52 |  | XKRY2; XKRY | 1.51 |  | TOB2 | 1.51 |
| NBN | 1.53 |  | TRHDE-AS1 | 1.52 |  | RAG1 | 1.51 |  | ULBP2 | 1.51 |
| SHOX | 1.53 |  | RBP5 | 1.52 |  | LOC339059; | 1.51 |  | SLC10A2 | 1.51 |
| MS4A4E | 1.53 |  | ANG; RNASE4 | 1.52 |  | RP5-1142A6.8 |  |  | GAPDH | 0.99 |
| KERA | 1.53 |  | C15orf53 | 1.52 |  | KIF18B | 1.51 |  | SOX9 | 0.96 |
| ACYP1 | 1.53 |  | KNSTRN | 1.52 |  | CDH19 | 1.51 |  | ACTB | 0.80 |
| INSR | 1.53 |  | TTC23 | 1.52 |  | MRPS12 | 1.51 |  | FABP7 | 0.67 |
| EIF2S2 | 1.53 |  | ACADVL | 1.52 |  | SPINT3 | 1.51 |  | S100B | 0.66 |
| CLIC6 | 1.53 |  | CCR7 | 1.52 |  | VN1R10P | 1.51 |  | GFAP | 0.46 |
| NR4A2 | 1.53 |  | POTEC | 1.52 |  | NAP1L2 | 1.51 |  |  |  |
| PARS2 | 1.53 |  | ZNF506 | 1.52 |  | SOX8 | 1.51 |  |  |  |
| C1orf52 | 1.53 |  | HKR1 | 1.52 |  | CA6 | 1.51 |  |  |  |
| ETV3 | 1.53 |  | MAMSTR | 1.52 |  | HSPA6 | 1.51 |  |  |  |
| UBR3 | 1.53 |  | LTN1 | 1.52 |  | SSFA2 | 1.51 |  |  |  |
| ESPNL | 1.53 |  | TAB1 | 1.52 |  | FAM178B | 1.51 |  |  |  |
| MLX | 1.53 |  | MRTO4 | 1.52 |  | KCTD8 | 1.51 |  |  |  |

Supplemental Table 1 (continued)
